# DFI-seq identification of environment-specific gene expression in uropathogenic *Escherichia coli*

**DOI:** 10.1186/s12866-017-1008-4

**Published:** 2017-04-24

**Authors:** Michelle Madelung, Tina Kronborg, Thomas Koed Doktor, Carsten Struve, Karen Angeliki Krogfelt, Jakob Møller-Jensen

**Affiliations:** 1 Department of Biochemistry and Molecular Biology, University of Southern Denmark, Campusvej 55, 5230 Odense M, Denmark; 2 Department of Microbiology and Infection Control, Statens Serum Institut, Artillerivej 5, 2300 Copenhagen S, Denmark

**Keywords:** DFI, NGS, UPEC, Amino acid biosynthesis, Virulence, UTI

## Abstract

**Background:**

During infection of the urinary tract, uropathogenic *Escherichia coli* (UPEC) are exposed to different environments, such as human urine and the intracellular environments of bladder epithelial cells. Each environment elicits a distinct bacterial environment-specific transcriptional response. We combined differential fluorescence induction (DFI) with next-generation sequencing, collectively termed DFI-seq, to identify differentially expressed genes in UPEC strain UTI89 during growth in human urine and bladder cells.

**Results:**

DFI-seq eliminates the need for iterative cell sorting of the bacterial library and yields a genome-wide view of gene expression. By analysing the gene expression of UPEC in human urine we found that genes involved in amino acid biosynthesis were upregulated. Deletion mutants lacking genes involved in arginine biosynthesis were outcompeted by the wild type during growth in human urine and inhibited in their ability to invade or proliferate in the J82 bladder epithelial cell line. Furthermore, DFI-seq was used to identify genes involved in invasion of J82 bladder epithelial cells. 56 genes were identified to be differentially expressed of which almost 60% encoded hypothetical proteins. One such gene *UTI89_C5139*, displayed increased adhesion and invasion of J82 cells when deleted from UPEC strain UTI89.

**Conclusions:**

We demonstrate the usefulness of DFI-seq for identification of genes required for optimal growth of UPEC in human urine, as well as potential virulence genes upregulated during infection of bladder cell culture. DFI-seq holds potential for the study of bacterial gene expression in live-animal infection systems. By linking fitness genes, such as those genes involved in amino acid biosynthesis, to virulence, this study contributes to our understanding of UPEC pathophysiology.

**Electronic supplementary material:**

The online version of this article (doi:10.1186/s12866-017-1008-4) contains supplementary material, which is available to authorized users.

## Background

Urinary tract infections (UTIs) are one of the most common infectious diseases and approximately 40–50% of women will experience a UTI in the course of their lifetime. Symptoms can range from mild irritative voiding, to bacteraemia, sepsis, and even death [1]. The economic burden is high and in 2010 it was estimated that the annual direct and indirect cost of UTIs in the United States was $2.3 billion. A wide range of bacterial species including *Klebsiella* spp., *Pseudomonas aeruginosa* and *Streptococcus agalactiae* are capable of causing UTIs. The majority of infections are however caused by uropathogenic *E. coli* (UPEC), which is responsible for 75% of all community-acquired UTIs [2].

UPEC bacteria encounter histologically distinct environments during their ascent through the urinary tract of humans. Colonization of the periurethral area is followed by bacterial entry into the bladder via the urethra. UPEC grow and persist in the bladder despite the constant urine flow they experience. During acute infection of the bladder, UPEC infect bladder epithelial cells (BECs) to initiate biofilm-like intracellular bacterial community (IBC) formation [3]. The acute infection ends with superficial BECs being exfoliated due to inflammation brought on by the invading bacteria [4]. At the same time, IBCs mature and the intracellular bacterial population display phenotypic variation; some cells become motile and rod-shaped while others turn into filaments more than 50 micrometer in length. At this stage the bacteria burst out into the lumen of the bladder [5]. The inflammation brought on by UPEC leads to recruitment of polymorphonuclear neutrophils (PMNs) into the bladder, which eliminates the majority of the released rod shaped UPEC. Filamentous bacteria resist clearance by the PMNs however, and are capable of reverting back to rod shape to initiate a second round of infection and IBC formation [6–8]. Bladder cell exfoliation renders underlying layers of undifferentiated tissue accessible to bacterial invasion. Inside the deeper tissue layers, UPEC can form quiescent intracellular reservoirs (QIRs), consisting of a single or few non-dividing bacteria. These QIRs may constitute a reservoir for recurrent UTIs [5, 9]. From the bladder, UPEC can further ascend the ureters to the kidneys where they trigger inflammation. Finally, in severe cases, UPEC may traverse into the bloodstream causing life-threatening sepsis [10].

Several virulence- or fitness-associated factors have been identified to be involved in UPEC infection of the urinary tract. Among these factors are adhesive fimbriae: type-1 fimbriae which bind to the urothelium of the bladder during acute infection [11], F9/Yde/Fml pili which are involved in specific binding to inflamed bladder tissue [12], and P fimbriae that are important during kidney infections [13]. Additional factors include the K1 capsule, which has a role in facilitating intracellular UPEC proliferation and IBC formation [14], the surface adhesin antigen 43, an autotransporter protein that promotes autoaggregation and is expressed by the bacteria embedded in the polysaccharide matrix of IBCs [3], iron acquisition genes necessary for bacterial growth in the iron limited environment of the host [15], and the toxins α-Hemolysin [16, 17], cytotoxic necrotizing factor type 1 [17] and secreted autotransporter toxin [18], all affecting the host urothelium during infection.

Recently, a redefinition of the concept of bacterial virulence was proposed [19]. It has been shown that UPEC fitness in the urinary tract depends on the tricarboxylic acid cycle and gluconeogenesis [20]. Moreover, peptide transporters have been shown to be induced in urine and to be required for fitness during infection [20]. Hence, virulence is determined by the sum of required metabolic pathways, the traditional virulence determinants, and upregulated transport systems and other indispensable functions [19]. In this study, we focus on environment-specific gene expression to further our understanding of UPEC pathophysiology. The experiments are centred on identification of genes involved in environment-specific adaptation, growth, and persistence during urine exposure in the bladder, and invasion and intracellular proliferation in the superficial cells of the bladder.

We combined the differential fluorescence induction (DFI) technique [21] with next-generation sequencing (NGS) to identify UPEC genes that are differentially expressed in response to human urine and growth inside the BEC line J82.

In DFI, a promoter trap library is created by inserting chromosomal DNA fragments from the bacteria of interest into a plasmid containing a promoterless *gfp* gene, encoding green fluorescent protein (GFP). The plasmids are then transformed into the bacteria of interest. The resulting promoter trap library is subjected to the conditions under investigation and to a reference condition. By performing consecutive fluorescence-activated cell sorting (FACS) rounds, activated promoters can be identified.

**Fig. 1:**
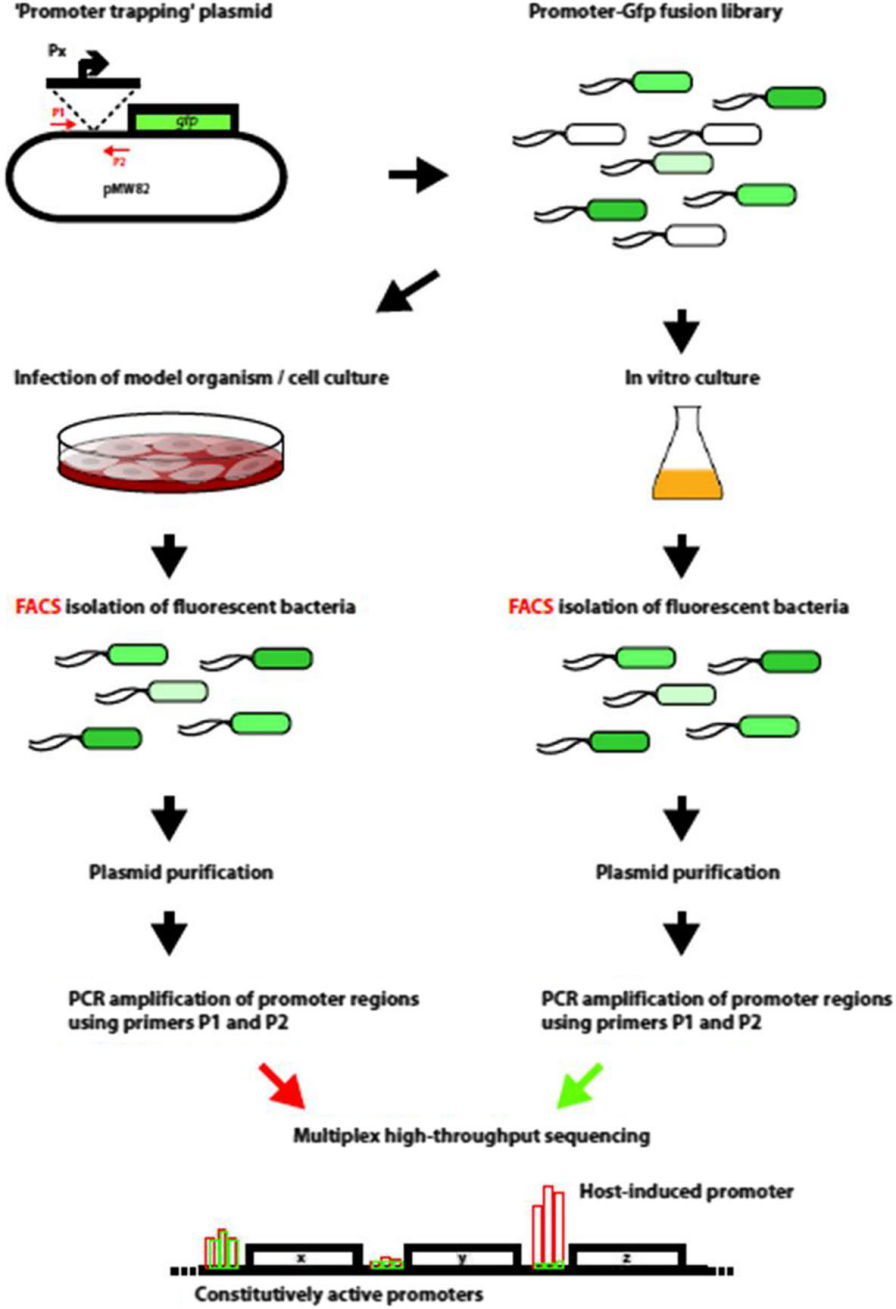
A promoter trap library is created using a *gfp* gene as reporter. The library is transformed into the bacteria of interest. The pool of transformants is both used to infect a tissue culture model and grown in vitro. From both environments, *green* fluorescent bacteria are enriched using a fluorescence-activated cell sorting instrument and grown over night in vitro. From the two populations plasmids are purified and PCR is performed to amplify trapped promoters. Through next-generation sequencing, host-induced promoters can be identified by comparing promoter read counts between the two conditions

Combining DFI with NGS (termed DFI-seq) is expected to enable a comprehensive and genome-wide view of differential gene expression compared to DFI alone [22], as the DFI-seq principle, as outlined in Fig. 1, eliminates the need for consecutive sorting of the bacterial library, thereby limiting potential biases associated herewith.

As with DFI, the bacterial library is exposed to two different environments- the environment under investigation, and a reference environment used to induce the expression of housekeeping genes. In DFI-seq however, the library is grown in parallel in the two respective environments, followed by isolation of fluorescent bacteria. The enriched genomic DNA contained in each promoter trap plasmid is then sequenced and mapped to the genome. By comparing the number of reads from each environment it is possible to identify promoters that are differentially expressed during growth in the two environments.

By applying DFI-seq on UPEC cultured in human urine we identify genes encoding hypothetical proteins as well as genes involved in amino acid biosynthesis to be differentially expressed in human urine. Five genes involved in arginine biosynthesis were found to be induced during growth in human urine. Deletion mutants of these genes exhibit low competition compared to the wild type during competition assays and demonstrated a reduced ability to invade or proliferate inside the BEC line J82. DFI-seq was further applied to UPEC infecting the BEC line J82 and resulted in the identification of 56 genes that were differentially expressed, including a previously uncharacterized gene *UTI89_C5139*, which was upregulated inside the bladder cells. Deletion of *UTI89_C5139* resulted in an enhanced ability to bind and invade or proliferate inside cultured BECs.

## Results

### Identification of urine-induced genes by DFI

Based on the physiological relevance and experimental tractability of human urine, we chose this microenvironment as the initial test condition for investigation into UPEC environment-specific gene expression.

As a reference for validation of DFI-seq, we first used the established DFI method to identify urine-induced genes in UTI89. In order to create a promoter trap library, genomic DNA from *E. coli* UTI89 was sheared into 500–700-basepair (bp) fragments and inserted upstream of a promoterless *gfp_ova* fusion reporter gene [23] on a medium copy-number plasmid (pMW82). The resulting plasmid library, termed pMWLib, was transformed into commercially available highly competent *E. coli* MegaX DH10B^TM^ T1^R^ Electrocomp^TM^ Cells, resulting in approximately 1.4×10^5^ independent transcriptional fusions, 80% of which carried an insert. This number corresponds to a seven-fold genome coverage [22, 24]. The plasmid DNA library was subsequently purified and used to transform *E. coli* UTI89, thereby giving rise to 4×10^6^ independent transformants. To verify that introduction of the plasmid library had no inhibitory effect on fitness, the DFI library was cultured in Lysogeny broth (LB) and compared to wild type UTI89. Consistent with previous reports [23], no growth inhibition was observed (data not shown).

UTI89/pMWLib was grown to mid-exponential phase in human urine followed by FACS-based enrichment of green-fluorescent bacteria. Next, the sorted bacteria were grown in LB followed by FACS enrichment of non-fluorescent bacteria. This two-step cycle was repeated to enrich for bacterial promoters that were selectively activated in human urine. After five consecutive sorts, the resulting bacteria were plated on LB medium to obtain clonal colonies. The percentage of green-fluorescent bacteria in the human urine-cultured bacterial population increased with every iteration; from 2 to 7.1 to 60.9% (Table 1).

**Table 1:** DFI sorting overview. Proportion of population enriched by sorting. Sort number 1, 3, and 5 enrich for green fluorescent bacteria; sort number 2 and 4 enrich for non-fluorescent bacteria

Sort number	Original bacterial culture	Growth medium	Proportion of population enriched by sorting
1	Original UTI89/pMWLib	Human urine	2% (*n* = 307,176)
2	Fluorescent bacteria isolated during the 1. Sort	LB	20.5% (*n* = 1,249,036)
3	Non-fluorescent bacteria isolated during the 2. Sort	Human urine	7.1% (*n* = 401,299)
4	Fluorescent bacteria isolated during the 3. Sort	LB	74.8% (*n* = 1,733,294)
5	Non-fluorescent bacteria isolated during the 4. Sort	Human urine	60.9% (*n* = 409,343)

We analysed the clonal heterogeneity of DFI-enriched bacteria by restriction fragment length polymorphism (RFLP) analysis. The RFLP patterns of 192 colonies revealed a high degree of clonal diversity (data not shown). PCR products amplified from the DNA inserts of 95 RFLP analysed colonies were therefore sequenced. The sequences could be mapped to 28 genes on the UTI89 genome using BLAST (http://blast.ncbi.nlm.nih.gov/) (Table 2).

**Table 2:** Sequenced inserts, gene name and protein product. UTI89 genome coordinates of identified inserts, and position relative to proximal genes. Colony identifiers are ordered in accordance to sequence start and end sites. Colonies marked in bold were used for GFP expression analysis. Differential fluorescence is reported as GFP fluorescence fold change of select UTI89/pMWLib clones grown in human urine versus LB

Gene	Protein	Gene start	Insert sequence start	Insert sequence end	Colony ID	Fold change in mean GFP signal
*argA*	N-acetylglutamate synthase	3139678	3139160	3139678	**8**, 57	13.5
			3139113	3139736	45	
*argC*	N-acetyl-gamma-glutamyl-phosphate reductase	4439034	4438574	4439108	**60**	8.2
			4438733	4439183	84	
*argE*	Acetylornithine deacetylase	4438919	4439100	4438541	**4**, 93	14.1
			4439305	4438787	42	
*argG*	Argininosuccinate synthase	3538472	3538296	3538904	51	4.3
			3538143	3538627	**82**	
*artJ*	Arginine-binding periplasmic protein 2	861650	862067	861578	**16**	7.9
*asnA*	asparagine synthetase A	4188023	4187539	4188150	**1**, 24, 38, 46, 49, 86	1.4
*asnB*	asparagine synthetase B	674199	674321	673791	30	Lower in urine
			674541	673908	35	
			674162	673571	**72**	
*manZ*	PTS system mannose-specific transporter subunit IID	1930575	1930463	1930677	**35**	Lower in urine
*ecnA*	entericidin A	4648116	4647814	4648373	**17**	2.1
*ecnB*	entericidin B	4648352				
*ilvC*	Ketol-acid reductoisomerase	4218632	4218651	4219116	20	4.5
			4218333	4218799	**83**	
*ilvG*	Acetolacetate synthase 2 catalytic subunit	4211428	4210930	4211421	**7**, 10	13.8
*metA*	Homoserine O-succinyltransferase	4462506	4462367	4462876	2	2.4
			4462142	4462760	9, 11	
			4462396	4462910	**34**, 52, 53, 66, 73, 79, 90	
			4462388	4463001	81	
*metC*	Cystathionine beta-lyase	3362694	3362313	3362827	**76**	2.7
*metE*	5-methyltetrahydropteroyltriglutamate-homocysteine S-methyltransferase	4277928	4277452	4277904	6, 26, 67	25.4
			4277357	4277878	**13**, 44, 58, 87	
			4277611	4278191	74	
*metF*	5,10-methylenetetrahydrofolate reductase	4416931	4416691	4417181	**18**, 23, 31	42.5
*potF*	putrescine ABC transporter substrate-binding protein	854834	854439	855001	**5**	2.4
			854460	855009	43	
*serA*	D-3-phosphoglycerate dehydrogenase	3236056	3236446	3236125	**41**	3.9
*yaiM*	hypothetical protein	395196	395661	395108		
*UTI89_C1129*	outer membrane heme/hemoglobin receptor	1122532	1122106	1122627	**91**	Lower in urine
*UTI89_C4885*	hypothetical protein	4787503	4787381	4787460		
*yajB*	ACP phosphodieterase	439176	439346	438767	**25**	Lower in urine
*ybdH*	hypothetical protein	617328	617543	617035	**3**, 15, 36, 54, 69, 85, 88, 92	9.5
			617447	617035	89	
			617636	617023	21, 50, 56, 77	
			617691	617083	28, 78	
			617543	617107	61	
*ybdL*	aminotransferase	617488	617206	617798	**33**, 37, 47	5.0
			617323	617815	62	
*yeaR*	Hypothetical protein	1912199	1912872	1912250	**65**	6.6
*yibI*	Hypothetical protein	4027446	4027775	4027295	**19**, 70, 75	5.6
			4027590	4027356	68	
Unannotated gene			48649	49106	**12**, 14, 22, 27, 32, 39, 40, 48, 55, 59, 64, 71, 80, 94, 95	Non-fluorescent
			48681	49177	63	
Opposite strand of *yagV*			315403	315898	**29**	Could not be cultivated in urine

16 genes were identified in two or more colonies. 13 genes were identified in two or more colonies containing different inserts, for details see Table 2. With the exception of three, all sequences identified in DFI mapped to regions upstream of genes.

Not all colonies could be unambiguously assigned to a unique chromosomal locus on the UTI89 genome. Sequences from four colonies each mapped upstream of two genes. The sequence from colony ID 17 mapped to a region spanning upstream of *ecnA*, the *ecnA* coding sequence and upstream of *ecnB*. The sequence from colony ID 35 mapped 634 nucleotides upstream of *asnB* and 215 upstream of *manZ*. Colony ID 91 mapped 522 nucleotides upstream of *UTI89_C1129* and 80 nucleotides upstream of *UTI89_4885*. Colony ID 41 mapped 554 nucleotides upstream of *yaiM* and 322 nucleotides upstream of *serA*.

Selected DFI-enriched colonies (marked in Bold in Table 2) were subsequently grown in human urine and LB to confirm the urine-specific transcriptional induction. The mean GFP fluorescence from these cultures was obtained by flow cytometry analysis, and the fold increase in GFP fluorescence is shown in Table 2. 19 colonies, representing 21 gene promoters, displayed fluorescence induction in human urine. The two cultures representing the *metE* and *metF* gene promoters showed highly induced GFP expression in human urine (25.4 and 42.5-fold, respectively). 12 cultures were upregulated between 3.9 and 14.1-fold, while five cultures showed less than 3-fold increase in GFP expression. The remaining six cultures, representing seven gene promoters, displayed either an increased GFP fluorescence level in LB compared to human urine, no fluorescence in human urine, or no growth in human urine. These are most likely false-positives resulting from erroneous inclusion in the sorted population.

**Fig. 2:**
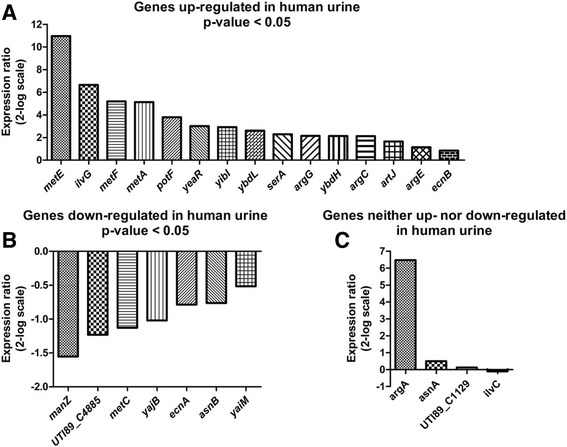
RT-qPCR verification of differentially expressed genes identified by DFI. RT-qPCR was performed on bacterial RNA isolated from UTI89 grown in human urine and LB, respectively. **a** and **b** Expression ratios (log2 scale) of genes differentially expressed in human urine compared to LB, with *P* < 0.05. **c** Gene expression ratios (log2 scale) of uniformly expressed genes (*P* > 0.05). A pairwise fixed reallocation randomisation test was performed [45] and exact *p*-values can be found in Additional file 10: Table S1

In order to confirm promoter induction, quantitative real time RT-PCR (RT-qPCR) analysis was employed on bacterial RNA isolated from UTI89 grown in human urine and LB. We analysed gene expression in the wildtype, testing promoter activity independent of the library clones. The region mapping to the pUTI89 plasmid, and the region mapping to the opposite stand of *yagV* was excluded from RT-qPCR, as GFP expression could not be verified for these regions. Of the 26 genes included in the RT-qPCR analysis, 15 genes were shown to be induced significantly in human urine whereas seven were expressed more strongly in LB compared to human urine. The four remaining genes could not be placed in either group (Fig. 2). Thus, out of 26 regions found by DFI to be differentially expressed during UTI89 growth in human urine compared to LB, 15 genes were confirmed by GFP expression analysis and RT-qPCR (Fig. 2a) to be upregulated.

### Identification of urine-induced genes by DFI-seq

We moved on to perform DFI-seq on UPEC cultured in human urine. UTI89/pMWLib was grown in human urine and LB in parallel followed by FACS-based isolation of green-fluorescent bacteria from both samples. In human urine, 2% of the population was found to be fluorescent; this percentage was 8.1 in LB. We sorted 307,176 events from the human urine culture and 1,315,930 events from the LB cultured sample. Sorted populations of green-fluorescent bacteria were cultured overnight for plasmid preparation, followed by PCR amplification of the inserted DNA segments. The PCR products were subsequently fragmented into 250–400 bp segments and prepared for single-end Illumina sequencing using an in-house protocol [25].

2,269,169 reads from the LB-grown sample and 1,042,702 reads from the human urine-grown sample were mapped to the UTI89 genome. The average read length was 67 bp in both samples and 31.53% of LB culture reads and 33.79% of the human urine culture reads uniquely mapped to the UTI89 genome. Promoters were defined as the region spanning from 300 bp upstream of the transcription start site (TSS) to 50 bp downstream of the TSS. Operons were defined as bookended transcripts, i.e. genes with no base pairs separating them. The number of reads mapping to a defined promoter was compared between the two growth conditions. 33 genes were identified to be upregulated in human urine compared to LB, i.e. with a positive regularized logarithm fold change (rLogFC) [26] of the genes or defined operons of at least 1.5. 31 genes were identified to be downregulated in human urine compared to LB, i.e. with a negative rLogFC of the genes or defined operons of −1.5 or less. Genes and defined operons with an absolute rLogFC of at least 1.5 are listed in Table 3 (upregulated genes) and Table 4 (downregulated genes).

**Table 3:** rLogFC values for genes upregulated in human urine. BLAST results of hypothetical proteins are represented in parentheses

Genes upregulated during growth in human urine compared to LB	Protein	Number of genes in operon	rLogFC
***ybdL***	Aminotransferase	1	2.453653
***ybdH***	Hypothetical protein (oxidoreductase)	1	2.351791
***metE***	5-methyltetrahydropteroyltriglutamate-homocysteine S-methyltransferase	1	2.285647
***metR***	DNA-binding transcriptional dual regulator for metE and MetH	1	2.236911
***yjaB***	Hypothetical protein (acetyltransferase)	1	2.137446
***metA***	Homoserine O-succinyltransferase	1	2.033752
***argC*** *;* *argB*	N-acetyl-gamma-glutamyl-phosphate reductase; Acetylglutamate kinase	2	2.026239
***metF***	5,10-methylenetetrahydrofolate reductase	1	2.010415
***argE***	Acetylornithine deacetylase	1	1.957286
***ilvY***	IlvY DNA-binding transcriptional dual regulator	1	1.947862
***argD***	bifunctional N-succinyldiaminopimelate-aminotransferase/acetylornithine transaminase	1	1.91199
***yibI***	Hypothetical protein	1	1.905502
***artJ***	Arginine-binding periplasmic protein 2	1	1.808872
***argG***	Argininosuccinate synthase	1	1.797071
***ilvG*** *;* *ilvM*	Acetolacetate synthase 2 catalytic subunit; acetolactate synthase 2 regulatory subunit	2	1.752627
***yifB*** *;* *UTI89_C4323*	predicted ATP-dependent protease; hypothetical protein	2	1.711417
***ydcX***	Hypothetical protein (membrane protein)	1	1.634447
*UTI89_C2996;* *UTI89_C2997;* *UTI89_C2998;* *yfdN1;* *UTI89_C3000*	bacteriophage V crossover junction endodeoxyribonuclease; hypothetical protein; bacteriophage V DNA adenine methylase; hypothetical protein; hypothetical protein	5	1.618326
***Crl***	DNA-binding transcriptional regulator Crl	1	1.554786
***potF***	putrescine ABC transporter substrate-binding protein	1	1.529378
***metJ***	transcriptional repressor protein MetJ	1	1.513308
***yibH***	Hypothetical protein (membrane protein)	1	1.511398
***UTI89_C2260***	Hypothetical protein	1	1.503588
***UTI89_C0254*** *;* *UTI89_C0255;* *UTI89_C0256*	Hypothetical protein; Hypothetical protein (peptidase C39); Hypothetical protein	3	1.502547

**Table 4:** BLAST results of hypothetical proteins are represented in parentheses

Genes downregulated during growth in human urine compared to LB	Protein	Number of genes in operon	rLogFC
***fliF*** *;* *fliG;* *fliH;* *fliI*	flagellar MS-ring protein; flagellar motor switch protein G; flagellar assembly protein H; flagellum-specific ATP synthase	4	2.177048
***fliE***	flagellar hook-basal body protein FliE	1	2.158562
***UTI89_C3136***	Hypothetical protein (FAD-linked oxidoreductase)	1	1.979745
*ybeK*	Hypothetical protein (hydrolase)	1	1.857649
***nadC***	quinolinate phosphoribosyltransferase	1	1.789156
***nagA***	N-acetylglucosamine-6-phosphate deacetylase	1	1.739815
***menE*** *;* *menC;* *menB*	O-succinylbenzoic acid-CoA ligase; O-succinylbenzoate synthase; naphthoate synthase	3	1.731787
***galS***	DNA-binding transcriptional regulator GalS	1	1.715122
***UTI89_C0374***	Hypothetical protein (nucleoprotein/polynucleotide-associated enzyme)	1	1.701595
***fadB***	multifunctional fatty acid oxidation complex subunit alpha	1	1.682517
***ampD*** *;* *ampE*	N-acetyl-anhydromuranmyl-L-alanine amidase; regulatory protein AmpE	2	1.671218
***narG*** *;* *narH;* *narJ;* *narI*	respiratory nitrate reductase 1 subunit alpha; respiratory nitrate reductase 1 subunit beta; nitrate reductase 1 subunit delta; nitrate reductase 1, cytochrome b(NR) subunit gamma	4	1.630201
***lacI***	lac repressor	1	1.604708
***pepQ*** *;* *yigZ*	Proline depeptidase; hypothetical protein	2	1.580097
***yiiM***	Hypothetical protein (6-N-hydroxylaminopurine resistance protein)	1	1.559754
***yjeM***	Transporter YjeM	1	1.536508
***purC***	phosphoribosylaminoimidazole-succinocarboxamide synthase	1	1.528491
***ynfK***	dithiobiotin synthetase	1	1.508037
***ybaS***	Glutaminase	1	1.507194
***hlyA***	hemolysin A	1	1.50293
*ybaR*	copper-transporting P-type ATPase	1	1.501551

**Fig. 3:**
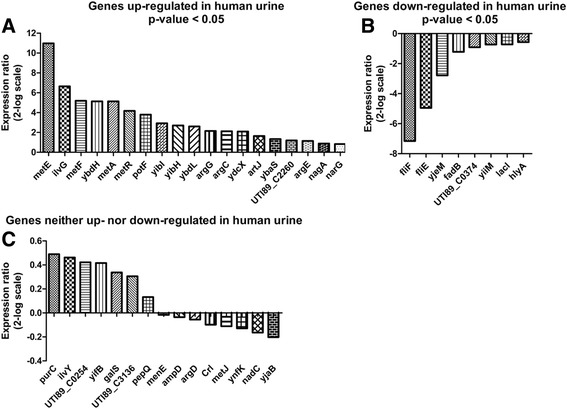
RT-qPCR verification of differentially expressed genes identified in the DFI-seq experiment. RT-qPCR was performed on bacterial RNA isolated from UTI89 grown in human urine and LB, respectively. **a** and **b** Expression ratios (log2 scale) of genes differentially expressed in human urine compared to LB, with *P* < 0.05. **c** Gene expression ratios (log2 scale) of uniformly expressed genes (*P* > 0.05). A pairwise fixed reallocation randomisation test was performed [45] and exact *p*-values can be found in Additional file 11: Table S2

The activity of identified promoters, was analysed by RT-qPCR using bacterial RNA isolated from UTI89 grown in human urine and LB, respectively. Figure 3 shows the expression ratio of all 42 genes analysed. Of the 23 genes identified with DFI-seq to be upregulated in human urine (marked in bold in Table 3), 16 were confirmed. Of the 19 genes downregulated in human urine (marked in bold in Table 4), eight were confirmed. Three genes (*ybaS*, *nagA* and *narG*) yielded inconsistent results as they were identified as upregulated in DFI-seq but downregulated by RT-qPCR. An explanation for this could be that regulatory elements of these promoters may have been omitted during the DFI library construction.

**Fig. 4:**
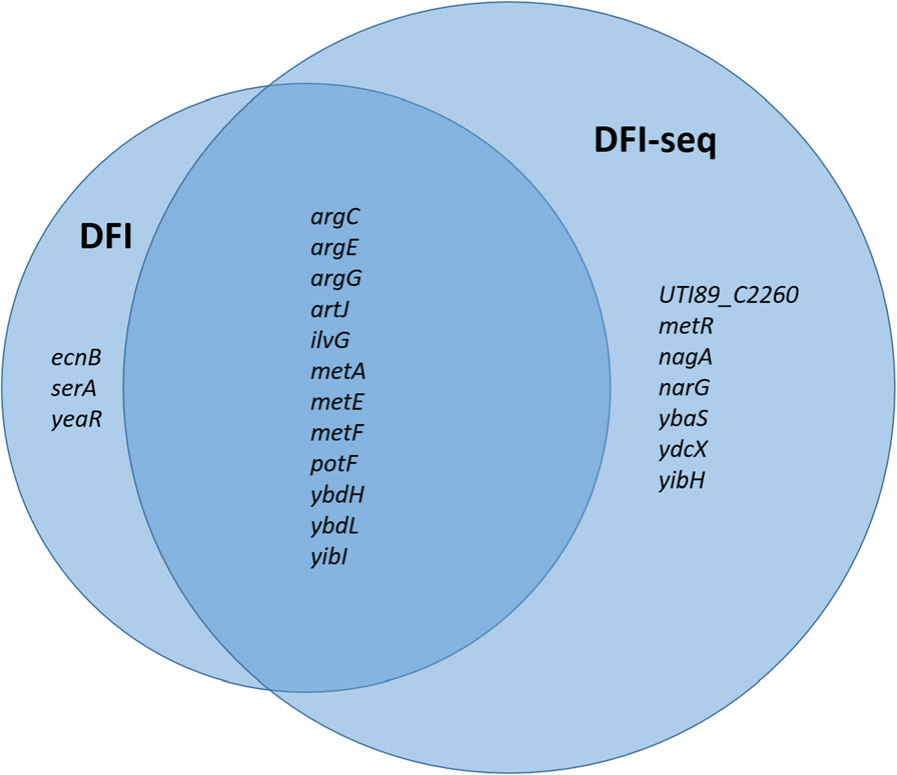
Area-proportional Venn-diagram demonstrating the overlap between DFI and DFI-seq results. Genes included were all confirmed by RT-qPCR

Twelve of the 15 genes identified by DFI were also identified in DFI-seq. In Fig. 4, a Venn diagram showing the extensive overlap of the DFI and DFI-seq results, is presented. DFI-seq yields a larger set of unique gene identifications compared to DFI. It further allows for identification of upregulated as well as downregulated promoters in the same experiment, thus providing a more comprehensive overview of changes in the transcriptional activity during bacterial growth in human urine vs. LB.

### Functional assessment of urine-induced UPEC genes

A total of 18 genes from both screens were selected for functional investigation. The 18 genes were deleted using the Lambda-Red-mediated recombination method devised by Datsenko and Wanner [27]. Two thirds of the selected genes are known to be directly or indirectly involved in amino acid metabolism, exceptions being *potF*, *ybdH*, *ybdL*, *yeaR*, *yibI* and *yjaB*. The *potF* and *ybdL* genes encode putrescine ABC transporter substrate-binding protein and an aminotransferase respectively; the remaining four genes all encode hypothetical proteins. By BLAST analysis these were predicted to encode an oxidoreductase (*ybdH*), proteins of unknown function (*yeaR* and *yibI*), and an acetyl transferase (*yjaB*).

**Fig. 5:**
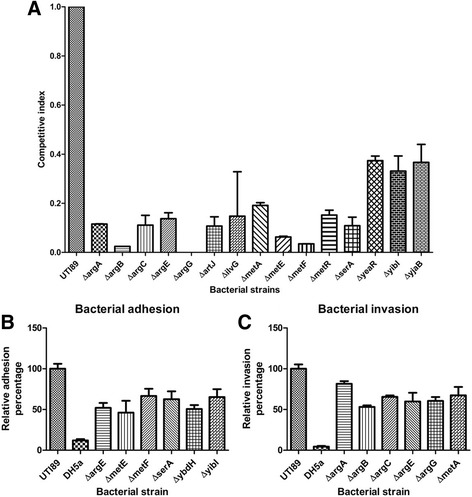
Fitness and relative cell adhesion and invasion of select deletion mutants. **a** Competitive index of deletion mutants during growth in human urine. The standard error of the mean of two independent 24-h growth experiments is shown (*P* < 0.05), the exact *p*-value can be seen in Additional file 7: Additional file 7: Table S3. **b** Mutants with statistically significant (*P* < 0,05) adhesion defects to J82 cells, the exact *p*-value can be seen in Additional file 9: Table S4. **c** Mutants with statistically significant (*P* < 0,05) invasion defects the exact *p*-value can be seen in Additional file 8: Table S5. The standard error of the mean of three independent assays is shown. **a, b** and **c** Unpaired t tests with two-tailed *p*-values were performed using GraphPad Prism 5 software

To assess the effect of deletion mutations on UTI89 fitness in human urine, competition assays were employed. The 18 mutant strains were grown in human urine in a 1:1 ratio with the UTI89 wild type. After 24 h, 15 of the mutants were significantly outcompeted to varying degrees (Fig. 5a).

All mutants affected in L-arginine biosynthesis were outcompeted during growth in human urine. The *argA*, *argB*, *argC*, *argE* and *argG* mutants had competitive indexes (CIs) of only 0.12, 0.02, 0.11, 0.14 and 0.000007, respectively. For genes involved in methionine biosynthesis the CIs were 0.19 (*metA*), 0.06 (*metE*), 0.03 (*metF*) and 0.15 (*metR*). Other genes directly or indirectly involved in amino acid metabolism were important for fitness, displaying CIs of 0.11 (*artJ* and *serA*) and 0.15 (*ilvG*). Mutants in *yeaR* or *yjaB*, two genes with unknown function, the CIs were 0.37 for both, and for the *yibI* mutant it was 0.33. Disruption of *potF*, *ybdH* and *ybdL* did not result in a statistically significant fitness defect (Additional file 1: Figure S1).

Urine is the environment encountered by UPEC before invasion of BECs and it is therefore reasonable to investigate whether the 18 genes might have an influence on the invasion or intracellular proliferation of UTI89 in BECs. The BEC-line J82 was infected with wild type UTI89 or mutant strains at a multiplicity of infection (MOI) of 100. After gentamicin-protection assays intracellular survival was evaluated. All null mutants affected in L-arginine biosynthesis showed an attenuated ability to invade BECs along with UTI89Δ*metA* (Fig. 5c). The remaining mutants showed no statistically significant difference in their ability to invade the J82 BECs (Additional file 2: Figure S2).

We investigated whether the reduction in intracellular bacterial counts of the *metA* and *arg* mutants was due to impaired BEC adhesion. Among the null mutants affecting L-arginine biosynthesis, only the Δ*argE* mutant displayed a reduced adhesion capacity. In addition to *argE*, the deletion of five other genes resulted in reduced adherence to BECs, *metE*, *metF*, *serA*, *ybdH*, and *yibI* (Fig. 5b). The remaining mutants showed no statistically significant difference in their ability to adhere to the J82 BECs (Additional file 3: Figure S3).

### Identification of BEC-induced UTI89 genes using DFI-seq

Having validated the use of DFI-seq in human urine we next moved on to employ the method on infected bladder cells. The J82 BEC line was infected with the UTI89 promoter trap library at an MOI of 10 and as control the library was grown simultaneously in DMEM media. The low MOI was used to minimize the incidence of single-cell invasion by multiple non-clonal bacteria. The two samples were subjected to FACS-based isolation of green-fluorescent BECs or bacteria, respectively.

In DMEM medium, 2.6% of the population was found to be fluorescent and in the BEC sample this percentage was 0.4%. From the BEC sample 4,320 events were sorted, whereas 193,105 events were sorted from the bacterial sample. 10,736,670 reads from the BEC sample and 14,820,780 reads from the bacterial sample were mapped to the UTI89 genome. The average read length was 50 bp in both samples, and the fraction uniquely mapping to the UTI89 genome was 60.93 and 69.65%, respectively.

The number of reads mapping to a defined promoter was compared between the two samples, leading to the identification of 34 promoter regions upregulated inside BECs with a positive rLogFC of at least 1.5. 16 promoter regions were identified to be downregulated inside BECs with a negative rLogFC of −1.5 or less. Genes and defined operons with an absolute rLogFC of at least 1.5 are listed in Table 5 (upregulated genes) and Table 6 (downregulated genes).

**Table 5:** UTI89 genes upregulated in J82 BECs. BLAST results of hypothetical proteins are represented in parentheses

Genes upregulated during growth in J82 BECs	Protein	Number of genes in operon	rLogFC
*trpR*	Trp operon repressor	1	2,332854642
*yaaU*	metabolite transport protein YaaU	1	2,283687208
*UTI89_C4979*	hypothetical protein (Transposase)	1	2,249895676
*UTI89_C4140*	hypothetical protein	1	2,15066842
*rluA*	23S rRNA/tRNA pseudouridine synthase A	1	2,07065298
*pepQ;* *yigZ*	Proline dipeptidase; hypothetical protein	2	1,975901548
*UTI89_C5123; UTI89_C5124*	tail component of prophage CP-933 K; tail component of prophage CP-933 K	2	1,86063772
*lplA*	lipoate-protein ligase A	1	1,852945008
*yceI*	hypothetical protein	1	1,840726794
*UTI89_C5140*	hypothetical protein	1	1,83941429
*UTI89_C5136*	hypothetical protein (putative membrane protein)	1	1,837077447
*UTI89_C5163*	hypothetical protein (transcriptional regulator)	1	1,72527562
*UTI89_C5093*	hypothetical protein (Phytoene synthase)	1	1,701749646
*yfdQ2*	hypothetical protein (phage protein)	1	1,699919702
*deoA*	thymidine phosphorylase	1	1,698786977
*UTI89_C5087; UTI89_C5088*	hypothetical protein (phage protein); hypothetical protein (HNH endonuclease)	2	1,689083782
*UTI89_C0227*	LysR family transcriptional regulator	1	1,666994808
*yjjJ*	hypothetical protein (transcriptional regulator)	1	1,659851375
*UTI89_C5141*	hypothetical protein	1	1,65493764
*yjjK*	ABC transporter ATP-binding protein	1	1,65080455
*deoB;* *deoD*	Phosphopentomutase; purine nucleoside phosphorylase	2	1,637364571
*UTI89_C5086*	hypothetical protein (putative membrane protein)	1	1,619322431
*yecH*	hypothetical protein	1	1,607068129
*yafD*	hypothetical protein (EEP domain-containing protein)	1	1,587086745
*UTI89_C5108*	hypothetical protein	1	1,572251909
*Slt*	lytic murein transglycosylase	1	1,55866374
*UTI89_C5139*	hypothetical protein	1	1,551006847
*UTI89_C5078*	hypothetical protein	1	1,539466639
*osmY*	periplasmic protein	1	1,536342614
*UTI89_C5146*	hypothetical protein (membrane protein)	1	1,529533673
*yjjU;* *yjjV*	hypothetical protein (phospholipase); deoxyribonuclease YjjV (metal-dependent hydrolase)	2	1,525815587
*dnaC*	DNA replication protein DnaC	1	1,525438036
*UTI89_C3195*	hypothetical protein	1	1,510078684
*yjjM*	hypothetical protein (GntR family transcriptional regulator)	1	1,504013167

**Table 6:** UTI89 genes downregulated in J82 BECs. BLAST results of hypothetical proteins are represented in parentheses. Genes located on the UTI89 plasmid are marked in bold

Genes downregulated during growth in J82 BECs	Protein	Number of genes in operon	rLogFC
***repB***	replication protein	1	2,011113132
*htgA*	positive regulator for sigma 32 heat shock promoters	1	1,890514455
***UTI89_P010***	hypothetical protein (iron permease/transporter (membrane protein))	1	1,861559188
*ribF*	bifunctional riboflavin kinase/FMN adenylyltransferase	1	1,859516233
*yaaY*	hypothetical protein	1	1,848940285
***ydiA***	hypothetical protein	1	1,753697215
*gyrB*	DNA gyrase subunit B	1	1,74780503
*rob*	right origin-binding protein	1	1,738152589
*yajK*	thiamine biosynthesis protein ThiI	1	1,638543917
*creA*	hypothetical protein (protein CreA (Catabolite regulation protein A))	1	1,635400678
*ytfB*	hypothetical protein (Opacity-associated protein A)	1	1,617894201
***UTI89_P053***	hypothetical protein	1	1,614324659
*yjjX*	NTPase	1	1,60329406
***UTI89_P144***	hypothetical protein (putative endonuclease)	1	1,577892223
*yccW*	hypothetical protein (ribosomal RNA large subunit methyltransferase)	1	1,516636602
***UTI89_P046; UTI89_P047***	hypothetical serine-threonine protein kinase hypothetical protein	2	1,513872693

Among the 16 downregulated regions, six were placed on the UTI89 plasmid (marked in bold in Table 6). One of the plasmid-encoded genes encodes a product with known function; *repB*, is involved in controlling plasmid replication. Of the ten downregulated chromosomally encoded genes, four encode hypothetical proteins. 23 out of 39 upregulated genes encode hypothetical proteins, ten of which could not be assigned by BLAST to a protein function or a cellular location.

**Fig. 6:**
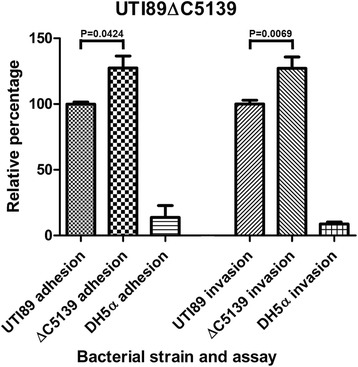
Cell adhesion and invasion of UTI89ΔC5139. The standard error of the mean of a minimum of three independent assays is shown for both adhesion and invasion. *P*-values are indicated above columns. An unpaired *t* test with two-tailed *p*-values was performed using GraphPad Prism 5 software

One gene was chosen for further investigation to show that validity of the method for identification of genes involved in invasion. *UTI89_C5139* was deleted from the UTI89 chromosome and tested for cell adhesion and invasion using the BEC-line J82 at an MOI of 100 (Fig. 6). The *UTI89_C5139* mutant displayed a significant increase of 25% in both cellular adhesion and invasion. The amino acid sequence (Uniprot; Q1R275-1) of the protein encoded by *UTI89_C5139* was analysed for presence of transmembrane helices using the TMHMM program (http://www.cbs.dtu.dk/services/TMHMM/) and was found to contain one transmembrane helix near the N-terminal suggesting the protein could be located in the outer membrane. Of the remaining 22 hypothetical genes found to be upregulated, four encode proteins that were found to contain transmembrane helices (Additional file 4: Table S6). Three of these proteins were by BLAST found to be putative membrane proteins (Table 5). UTI89_C5136, UTI89_C5146 and UTI89_C5068 have four, two and four transmembrane helices respectively. The remaining gene *UTI89_C5140* encodes a protein which contains a single transmembrane helix located near the N-terminal. *UTI89_C5140* is located 53 bases downstream from *UTI89_C5139* on the same strand, suggesting that these two genes might be transcribed simultaneously during infection.

## Discussion

The knowledge about UPEC fitness and virulence gene expression in the urinary tract is far from complete and with the emergence of multidrug-resistant (MDR) UPEC strains, limiting the available treatment options, new strategies are necessary to prevent UTIs [28]. A deeper understanding of UPEC adaption to the urinary tract, its fitness and pathogenicity would help in the search for new treatment or prevention options.

Gene expression is usually investigated by microarray analysis or, in recent years, by RNA-seq. By measuring specific RNA levels, both methods can give a genome-wide view of gene expression in a bacterial population. Both methods have been used on urine samples from patients with UTIs [29, 30]. However, when investigating infected tissue limitations arise. RNA purification of mixed samples results in an overwhelming amount of eukaryotic RNA that will diminish the information content that can be derived from the bacterial RNA by conventional approaches [31].

Another way to study environment-specific gene induction is by screening promoter trap libraries, which circumvents the need for RNA purification. It can be done using a conditionally essential reporter gene as done in in vivo expression technology (IVET) or a fluorescence marker as done in DFI [22]. IVET identifies genes that are constitutively and highly expressed. However, this method fails to detect transiently or weakly expressed genes, a limitation that was overcome with the introduction of the DFI method [32]. DFI and DFI-seq are not biased by the absolute expression levels of the selectable marker, and weakly and highly fluorescent clones are therefore sorted with equal efficiency. The DFI strategy is based on consecutive rounds of FACS based on differential GFP expression. The *gfp* gene has been replaced by a *gfp_ovalbumin* (*gfp-ova*) gene fusion [22], which encodes a destabilized GFP-Ovalbumin reporter fusion with less impact on bacterial fitness. This also enables examination of the active promoters in an environment at a specific time point, giving a snapshot of the activated genes. Both IVET and DFI only identify upregulated genes and eliminate housekeeping genes from the promoter trap library. In DFI this is achieved during the consecutive sorts. These consecutive sorts are time consuming and can introduce bias, however too few sorts can give to many false positive gene identifications.

In the previous DFI studies conducted by Valdivia et al. [21, 32] only three consecutive sorts were used. Here we used five sorting steps in order to reduce the number of false-positives (Table 1). Of the 25 clones picked for the confirmation of urine-specific GFP induction, 76% had a higher fluorescence in human urine compared to LB (Table 2). The induction of gene promoters measured by GFP-Ovalbumin protein, was confirmed using RT-qPCR. However, the increase in mean GFP fluorescence (Table 2) does not accurately reflect the change in mRNA level for a particular promoter (Fig. 2). During insertion of DNA fragments into the promoter trap plasmid, we may inadvertently have altered promoter activity, for example by excluding transcription factor binding sites from the inserted fragment.

By introducing DFI-seq, we attempt to simplify the DFI method and at the same time harness the power of NGS to generate genome-wide rather than clonal data. DFI-seq eliminates the need for consecutive sorting of the bacterial library, and thereby limits the bias that can be introduced. The method however can introduce bias due to overnight growth of the sorted bacteria, as with DFI, and when PCR amplifying the promoters before NGS. However, the method can identify both up- and downregulated genes in a single sorting step. The data from DFI and DFI-seq largely overlap, thus validating the use of the DFI-seq method over traditional DFI. By reducing bias DFI-seq diminishes the chance for generation of false-positive identifications. To increase the stringency of DFI-seq experiments and reduce the chances of generating false positives due to the biases that might be introduced, biological replicates should be included in the DFI-seq experiments. However, the possibility of excluding transcriptional factor binding sites still exist and DFI-seq should therefore be considered mainly as a qualitative discovery tool that requires RT-qPCR for quantitation of individual promoter outputs.

Human urine contains few amino acids and these serve as the main carbon source for bacteria growing in the urinary tract [33]. Biosynthesis of amino acids can therefore be considered a fitness factor for UPEC during UTI. Our results support this view, as 55% of the 22 genes confirmed by RT-qPCR to be upregulated in human urine (Figs. 2 and 3) were directly or indirectly involved in amino acid biosynthesis.

Serine and methionine biosynthesis have previously been reported to be necessary for optimal fitness of UPEC in human urine [34] and our observations were in accordance to this. The *serA* gene encodes D-3-phosphoglycerate dehydrogenase. This enzyme uses NAD^+^ in the conversion of 3-phospho-D-glycerate to 3-phosphohydroxypyruvate as a first step in serine biosynthesis [35]. In RT-qPCR the *serA* gene had an expression ratio of 2.3 (Fig. 2a), corresponding to an almost five-fold upregulation during growth in human urine compared to LB. This is in agreement with the findings by Alteri et al., which identified differentially expressed proteins in UPEC strain CFT073 during growth in human urine and iron-limited LB by comparative proteomics [20]. The *serA* null mutant was inhibited in its ability to adhere to J82 BEC (Fig. 5b) by about 60% compared to the wildtype, and displayed a CI of 0.11 during growth in human urine (Fig. 5a). We did not detect a significant difference between wildtype UTI89 and UTI89Δ*serA* during invasion of BEC-line J82. Alteri et al. showed that a *serA* mutant of UPEC strain CFT073 was not significantly out-competed by wild type CFT073 in vivo 48 h post infection of mice [20] and the mutant suffered only a slight fitness defect. These results taken together suggest that the effect of the *serA* gene is mainly related to growth in urine prior to host cell invasion.

Genes involved in methionine biosynthesis, *metA*, *metE*, *metF* and *metR* genes were found to be upregulated in human urine (Fig. 3a), while *metC* was downregulated (Fig. 2b). The *metA*, *metC* and *metE* genes encode enzymes involved in methionine biosynthesis; *metE* is positively regulated by the MetR DNA-binding transcriptional dual regulator. The *metF* gene is indirectly involved in methionine biosynthesis. MetF is involved in the biosynthesis of 5-methyltetrahydrofolate, which acts as a substrate for methylation in the last step of methionine biosynthesis in a reaction catalysed by MetE. Deletion mutants of *metA*, *metE*, *metF* and *metR* were all less competitive than the wild type during growth in human urine. The *metE* and *metF* mutants displayed reduced adhesion to J82 BECs (Fig. 5a), while the *metA* mutant was inhibited in its ability to invade or proliferate intracellularly in J82 BECs (Fig. 5b). Thus, our results are consistent with the findings of Hull and Hull [34] in showing that Methionine biosynthesis is important for UPEC fitness in urine and that mutations affecting this process can affect the virulence of UPEC, thereby connecting fitness genes to virulence.

Two additional amino acids, whose biosynthesis is important during growth in human urine, are isoleucine and arginine [36]. We found that the *ilvG* mutant was less competitive than the wild type (CI = 0,15) and that *ilvG* was upregulated during growth in human urine (Expression ratio (log2) of 6.65). The *ilvG* and *ilvM* genes encode the catalytic and regulatory subunit of acetolactate synthase 2, respectively. This enzyme uses pyruvate as a substrate during isoleucine biosynthesis. We did not detect differential expression of *ilvC*, which encodes another enzyme in isoleucine biosynthesis, was differentially regulated in human urine compared to LB (Fig. 2c). This gene has been reported by Snyder et al. to be upregulated in UPEC strain CFT073 during growth in human urine [36].

We analysed deletion mutants in six genes involved in arginine metabolism. The *artJ* gene encodes the Arginine-binding periplasmic protein 2, which is part of the Arginine ABC transport system. This system is composed of five proteins encoded by the *artP*, *artI*, *artQ*, *artM* and *artJ* genes, all located in succession on the UTI89 chromosome. The *artJ* null mutant was outcompeted in human urine by the wild type (CI = 0,11), however it was not significantly compromised in is ability to adhere to and invade or proliferate inside J82 BECs. Consistent with our findings, *artJ* was previously shown to be upregulated during growth on human urine agar plates [37], and upregulated in human urine [36].

Of the five *arg* genes investivated *argC*, *argE* and *argG* was confirmed by RT-qPCR to be upregulated. *argG* was previously found to be up-regulated in human urine compared to iron-limited LB [20], and a DNA microarray study on UPEC strain CFT073 found *argB*, *argC* and *argE* to be upregulated in human urine [36]. All mutants affected in L-arginine biosynthesis were outcompeted during growth in human urine (Fig. 5a), consistent with previous findings [34]. In urine, arginine is present at a suboptimal level for bacterial growth [34]. This could be the reason behind this requirement for endogenous synthesis of arginine in this extracellular environment. Arginine biosynthesis mutants were all inhibited in their ability to invade or proliferate inside J82 BECs (Fig. 5c) but only the *argE* mutant displayed reduced adhesion ability (Fig. 5b). CFT073Δ*arG* has been shown to resist out-competiton by CFT073 wild type in vivo 48 h post infection of mice [20]. The results however indicated a slight fitness defect in the kidneys but not the bladder. Another study showed that an *argC* mutant had a significantly decreased ability to infect the mouse kidney [38]. Taken together these results demonstrate a significant link between amino acid biosynthesis and virulence, and hence support the recent bacterial virulence paradigm suggested by Mobley [19]. Using growth competition assays, we show that the *arg* genes contribute to the fitness of UPEC in urine. However, these genes also play a role in the intracellular UPEC proliferation without affecting their initial adhesion to host cells. Thus, the *arg* genes are not only fitness factors but also virulence determinants enabling IBC formation - a charateristic of UPEC pathogenesis. The capacity for biosynthesis of specific amino acids may become critical during intracellular bacterial growth. In support hereof, the amino acid requirements of *Salmonella enterica* for intracellular proliferation have been shown recently to vary according to the infected cell type [39].

Yet another gene found in our study links back to arginine and methionine, *potF*. The *potF* gene encodes a substrate-binding protein, which is part of the ABC transporter of the polyamine putrescine. The predominant polyamines in bacteria are putrescine and spermidine. Arginine can be converted into putrescine and further into Spermidine [39]. Polyamines were found to be important for virulence in *Salmonella* as a polyamine mutant of *Salmonella* Typhimurium had a reduced intracellular survival/replication compared to the wild type and was attenuated in the mouse model of typhoid fever [39]. Thus, polyamines may serve as environmental cues for the bacteria to initiate virulence gene expression [39]. UPEC *potF* has been shown by microarray analyses to be downregulated in response to increased environmental osmolality caused by high salt and urea [40] and when isolated from the urine of infected women [30]. Our data showed that *potF* was upregulated in human urine compared to LB, but deletion had no effect on cell invasion. The ABC transporter affected in the *potF* deletion mutant is not the only ABC transporter capable of transporting putrescine, possibly explaining why we do not observe an effect on UPEC virulence in the *potF* mutant.

To demonstrate the usefulness of DFI-seq in a more complicated experimental setup, BECs were infected with UPEC strain UTI89 and subjected to DFI-seq. Of the 56 genes found to be differentially expressed in the BECs, almost 60% encoded hypothetical proteins. These may possibly represent virulence factors not yet discovered. To validate the DFI-seq method one gene was chosen for further investigation. The *UTI89_C5139* gene encodes a putative membrane protein, which contains a transmembrane domain. The deletion mutant exhibited increased adherence to J82 BECs and increased invasion or intracellular proliferation. *UTI89_C5139* was found during DFI-seq to be upregulated during invasion, and a deletion mutant of this gene was expected to be less invasive in ex vivo experiments. The opposite was observed, indicating that the gene product directly or indirectly influences the expression of other virulence genes, possibly limiting their effect to avoid immune system detection or a to high infection rate that might lead to premature exfoliation of the bladder lining both resulting in a lowering of the bacterial burden.

## Conclusion

DFI-seq allowed the positive selection of genes involved in UPEC growth in vitro in human urine as well as during infection of bladder tissue culture. DFI-seq provides a more comprehensive overview of differential gene expression than conventional DFI, it is less time-consuming and labor-intensive and has fewer handling steps that may introduce bias. We identified genes involved in amino acid metabolism necessary for growth in human urine compared to growth in LB by using DFI-seq. The arginine biosynthesis genes were found to be required for successful infection of BECs, thus strengthening the assertion that fitness and virulence gene expression are both important in UPEC pathogenicity. DFI-seq provides a new avenue for virulence and fitness gene identification using positive selection and we show that it can be used not only with liquid culture but also in cell culture assays. In order to firmly establish a definite role in virulence of the genes identified in this study, animal infection experiments are required. Thus, DFI-seq should be applicable to in vivo infection systems, thus providing important insights into host-induced changes in gene expression of infecting bacterial pathogens.

## Methods

### Bacterial strains, cells, and growth conditions

The UPEC strain UTI89, a cystitis-derived isolate [5] was used for library construction. UTI89-derived deletion mutants (Table 7) were constructed using the lambda Red recombination [27]. Primers used for mutant construction can be found in Additional file 5: Table S7. Additionally, *E.coli* DH5α was used as a non-pathogenic control strain.

**Table 7:** Bacterial strains and plasmids used in this study

Strain	Genotype	Source
UTI89	Serotype O18:K1:H7	[5] Provided by Emile Van Schaftingen
UTI89/pMW82	Amp^R^	This study
UTI89/pMWLib	Amp^R^	This study
DH5α	F– Φ80*lac*ZΔM15 Δ(*lac*ZYA-*arg*F) U169 *rec*A1 *end*A1 *hsd*R17 (rK–, mK+) *pho*A *sup*E44 λ– *thi*-1 *gyr*A96 *rel*A1	Invitrogen
UTI89Δ*argC*	Δ*argC*, Cml^R^	This study
UTI89Δ*argE*	Δ*argE*, Cml^R^	This study
UTI89Δ*argG*	Δ*argG*, Cml^R^	This study
UTI89Δ*artJ*	Δ*artJ*, Cml^R^	This study
UTI89Δ*ilvG*	Δ*ilvG*, Cml^R^	This study
UTI89Δ*metA*	Δ*metA*, Cml^R^	This study
UTI89Δ*metE*	Δ*metE*, Cml^R^	This study
UTI89Δ*metF*	Δ*metF*, Cml^R^	This study
UTI89Δ*potF*	Δ*potF*, Cml^R^	This study
UTI89Δ*ybdH*	Δ*ybdH*, Cml^R^	This study
UTI89Δ*ybdL*	Δ*ybdL*, Cml^R^	This study
UTI89Δ*yibI*	Δ*yibI*, Cml^R^	This study
UTI89Δ*argA*	Δ*argA*, Cml^R^	This study
UTI89Δ*serA*	Δ*serA*, Cml^R^	This study
UTI89Δ*yeaR*	Δ*yeaR*, Cml^R^	This study
UTI89Δ*argB*	Δ*argB*, Cml^R^	This study
UTI89Δ*metR*	Δ*metR*, Cml^R^	This study
UTI89Δ*yjaB*	Δ*yjaB*, Cml^R^	This study
UTI89Δ*C5139*	Δ*C5139*, Kan^R^	This study
Plasmid	Genotype	Source
pKD3	Cml template plasmid	[28]
pKD4	Kan template plasmid	[28]
pKD46	Bla, λ Red recombinase expression plasmid	[28]
pMW82		[22] Kindly provided by Dirk Bumann
pMWLib	pMW82 containing random 500−700 bp long UTI89 DNA segments	

Bacterial cultures were grown at 37 °C in LB medium, DMEM + 10% FBS, or human urine (USG 1.021) containing 30 μg/mL chloramphenicol (cml), 50 μg/mL kanamycin (kan) or 100 μg/mL ampicillin (amp) for plasmid selection. Human urine was prepared for experiments as described previously by Andersen et al. [41].

J82 BECs (ATCC® HTB-1™) were maintained at 5% CO_2_, as a monolayer in DMEM (ThermoFisher Scientific) containing 10% (v/v) heat-inactivated fetal bovine serum (FBS) (Gibco) and Penicillin (100 units/ml)-Streptomycin (100 μg/ml) (Gibco).

### Library construction

The UTI89 promoter trap library was constructed as described by Bumann and Valdivia [22] with minor modifications. Chromosomal DNA was purified from an overnight culture of UTI89. Bacterial cells were harvested and resuspended in TE buffer (0.089% Trizma hydrochloride (w/v), 0.053% Trizma base (w/v) and 0.1 mM EDTA), rapid lysis buffer (5% sodium dodecyl sulfate (SDS), 0.125 M EDTA, 0.5 M Tris–HCl pH 9.4), Ribonuclease A (Sigma Aldrich) and protease (Roche) (37 °C) and incubated for 1 h at 37 °C. Chromosomal DNA was purified by phenol-chloroform extraction, precipitated by addition of 2 vol. 96% ethanol, and resuspended in TE-buffer.

Purified genomic DNA (100 ng/ml) was disrupted by sonication for 2×30 s using a Branson Sonifier 250 (Branson Ultrasonics). Resulting fragments of 500–700 bp were purified from a 2% agarose gel using a GFX PCR DNA and Gel Band purification kit (GE Healtcare) and treated with T4 polymerase (New England Biolabs) for 15 min at 12 °C to repair single-stranded regions. The library vector pMW82 was cleaved with BamHI (Fermentas) and SphI (Fermentas), and treated with T4 polymerase to fill in single-stranded overhangs. Next, treatment with shrimp alkaline phosphatase (USB Corporation) was included to prevent vector re-ligation. Finally, genomic fragments and vector DNA was ligated using T4 DNA ligase.

Ligated DNA was introduced into MegaX DH10B^TM^ T1^R^ Electrocomp^TM^ Cells (Invitrogen) by electroporation, and transformants were selected by plating on LB agar containing 100 μg/ml amp. The frequency of vector re-ligation was estimated to be less than 20%. Plasmids were subsequently purified using NucleoBond AX kit (Macherey-Nagel) and transformed into *E. coli* UTI89 by electroporation. The resulting amp-resistant colonies, together constituting the promoter trap library UTI89/pMWLib, were pooled and frozen at −80 °C.

### Sorting and FACS analysis of bacterial cells

Bacterial cells were grown to mid-exponential phase (4–5 generations) at 37 °C with aeration in preheated LB or human urine (USG 1.021) with 100 μg/ml ampicillin or for 1 h without aeration in preheated DMEM with 10% FBS. For DFI analysis of UTI89/pMWLib, bacteria grown in urine were sorted based on green fluorescence and bacteria grown in LB were sorted based on the absence of green fluorescence. In DFI-seq, fluorescent bacteria were sorted for all growth conditions, LB, human urine and DMEM. The bacteria were then collected by centrifugation at 3500xg for 10 min, resuspended in LB and grown overnight.

### Sorting and FACS analysis of BECs

J82 grown to confluence in 15 cm dishes or 6-well plates were infected at an MOI of 10 for 1 h at 37 °C. The cells were then washed three times with PBS to remove non-invading bacteria and incubated in DMEM with 10% FBS and 100 μg/ml gentamicin for 2 h. Next, infected cells were washed three times with PBS to remove the gentamicin before 0.25% trypsin-EDTA was added for 5 min to release the cells into suspension. The cells were collected by centrifugation at 150 ×g and 4 °C for 10 min and resuspended in PBS containing 10% FBS.

J82 cells emitting green fluorescence were collected by cell sorting, using a FACSAria II. In order to preserve the samples, cell sorting was performed for a maximum of 1 h per sample, so several infection assays were set up to allow for successive sorting of multiple samples. Finally, bacteria were released from the J82 cells by addition of 1% Triton X-100 and vortexing for 60 s. The bacteria were then collected by centrifugation at 3500×g for 10 min, resuspended in LB and grown overnight.

### Sequencing and analysis of sequence data after DFI

Following FACS-based enrichment, the bacterial cells were plated on LB agar with 100 μg/ml amp and grown overnight at 37 °C. Resistance Fragment Length Polymorphisms (RFLP) patterns [22] were analysed by agarose gel separation. 95 colonies were sequenced using primers P1 (5′-TGAAGGCTCTCAAGGGCATC-3′) and P2 (5′-GTGTTGGCCATGGAACAGGT-3′). Sequences were aligned, using BLAST, with the *E. coli* UTI89 chromosome and pUTI89 sequences (NCBI database accession number NC007946 and NC007941). Annotation of fragments was performed using Ecocyc.org [42].

### Quantitative real time RT-PCR

Primers for RT-qPCR listed in Additional file 6: Table S8 were designed using Primer3 software [43, 44]. Total RNA was isolated by phenol/chloroform extraction from UTI89/pMW82 grown to mid-exponential phase (4–5 generations) at 37 °C with aeration in LB and human urine (USG 1.021), respectively. 1 μg of total RNA was treated with DNase I for 30 min at 37 °C, followed by heat inactivation of the enzyme. Purified RNA was then used for cDNA synthesis using the Maxima first strand cDNA synthesis kit (Thermo Scientific). Real time quantification was performed using SYBR® Select Master Mix (Life Technologies) as specified by the manufacturer. RT-qPCR reactions were run in 96-well plates in an Mx3005P QPCR System (Agilent Technologies) using *rrsA* and *rpoB* mRNA as internal controls. Measurements were performed in at least three technical replicates to enable determination of the relative fold change in mRNA expression between UTI89 grown in urine and LB [45].

### Sequencing and analysis of sequence data after DFI-seq

Plasmids were purified from sorted UTI89/pMWLib using NucleoBond PC 500 kit (Macherey-Nagel) as specified by the manufacturer. PCR amplification of the inserted chromosome segments in pMWLib was performed using primers P1 (5′-TGAAGGCTCTCAAGGGCATC-3′) and P2 (5′-GTGTTGGCCATGGAACAGGT-3′). For Illumina sequencing, the DNA fragments were sonicated into 250–400 bp long segments from the original size of 500–700 bp, and sequencing library preparation was performed according to the manufacturer’s instructions (Illumina) as described previously [25]. The library was sequenced using the Illumina HiSeq 1500 instrument.

Reads were aligned using STAR version 2.3.1z_r395 with spliced mapping disabled. Subsequently, uniquely aligning reads overlapping operon promoters were counted, and DESeq2 was used to identify promoters that were utilized differentially in the urine sample. The rLogFC was calculated. A positive rLogFC means that more reads map to a particular operon promoter region in the sample grown in human urine compared to LB. The opposite is true of a negative score. Operon promoters were defined as the region spanning from 300 bp upstream of the transcription start site to 50 bp downstream. Operons were defined as bookended transcripts, i.e. genes with no base pairs separating them.

### Competitive index

The fitness of single-gene deletion mutants was compared to that of the wild type in a growth competition assay. Cultures grown overnight with aeration in LB, washed in PBS and mixed in a 1:1 ratio (wild type:mutant) in human urine (USG 1.021). Bacteria were plated on LB agar with- and without chloramphenicol at the beginning of the experiment and after 24 h in order to determine the ratio of wild type versus mutant. The competitive index is defined as the output ratio of CFU of mutant to wild type bacteria divided by the input ratio of CFU of mutant to wild type bacteria. Thus, if a mutant strain is as competitive as its isogenic wild type parent, a value of 1 will be achieved, indicating that the mutant is not attenuated.

### Adhesion and infection assay

J82 were seeded into 24-well plates and grown to confluence at which point they were infected at an MOI of 100. The cells were washed three times in PBS to remove the antibiotic-containing DMEM and incubated for 1 h in prewarmed DMEM before addition of bacteria. The plates were centrifuged at 500 xg for 5 min to synchronize infection, and incubated for 1 h at 37 °C. The cells were then washed three times with PBS to remove non-adherent bacteria. To measure bacterial adherence, J82 cells were lysed by addition of 100 μl 0.25% trypsin-EDTA and 400 μL 0.1% Triton X-100 and plated onto LB agar. Bacterial invasion was measured using a gentamicin protection assay; after PBS wash, infected J82 cells were incubated in DMEM with 10% FBS and 100 μg/ml gentamicin for 2 h followed by washing, lysing and bacterial plating as described above.

### Statistics

For RT-qPCR analysis, pairwise fixed reallocation randomisation test was performed [45]. Data from growth competition, adhesion and invasion assays were analysed by unpaired t tests with two-tailed p-values using GraphPad Prism 5 software.

## Additional files

Additional file 1: **Figure S1.** Mutants with no growth defect. The standard error of the mean of two independent 24-h growth experiments is shown. The exact p-value can be seen Additional file 7: Table S3. Unpaired t tests with two-tailed p-values were performed using GraphPad Prism 5 software. (JPG 1007 kb)

Additional file 2: **Figure S2.** Mutants with no cell invasion defect. The standard error of the mean of three independent invasion assays are shown, *P* > 0.05 (the exact p-value can be seen in Additional file 8: Table S5). Unpaired t tests with two-tailed p-values were performed using GraphPad Prism 5 software. (JPG 3856 kb)

Additional file 3: **Figure S3.** Muatnts with no cell adhesion defect. The standard error of the mean of three independent invasion assays are shown, *P* > 0.05 (the exact *p*-value can be seen in Additional file 9: Table S4). Unpaired t tests with two-tailed p-values were performed using GraphPad Prism 5 software. (JPG 3905 kb)

Additional file 4: **Table S6.** TMHMM analysis of upregulated hypothetical genes. Gene name of hypothetical genes from Table 5, in parenthesis are the protein BLAST results. (DOCX 13 kb)

Additional file 5: **Table S7.** Primers used for lambda Red recombination. Up and Down primers are used when checking placement of the resistance gene. KO1 and KO1 are used for PCR of the resistance gene before the knockout reaction. (DOCX 15 kb)

Additional file 6: **Table S8.** Primers used for quantitative real time RT-PCR (DOCX 15 kb)

Additional file 7: **Table S3.** Competitive index *p*-values. (DOCX 12 kb)

Additional file 8: **Table S5.** *P*-values for cell invasion assays. (DOCX 12 kb)

Additional file 9: **Table S4.** *P*-values for cell adhesion assays. (DOCX 12 kb)

Additional file 10: **Table S1.** *P-*values for RT-qPCR verification of genes identified by DFI. (DOCX 12 kb)

Additional file 11: **Table S2.** *P*-values for RT-qPCR verification of genes identified by DFI-seq. (DOCX 13 kb)
