# Supplementary figures and images for: DFI-seq identification of environment-specific gene expression in uropathogenic Escherichia coli
Source: BMC Microbiol. 2017 Apr 24;17:99. doi: 10.1186/s12866-017-1008-4 (PMC5404293; doi:10.1186/s12866-017-1008-4)

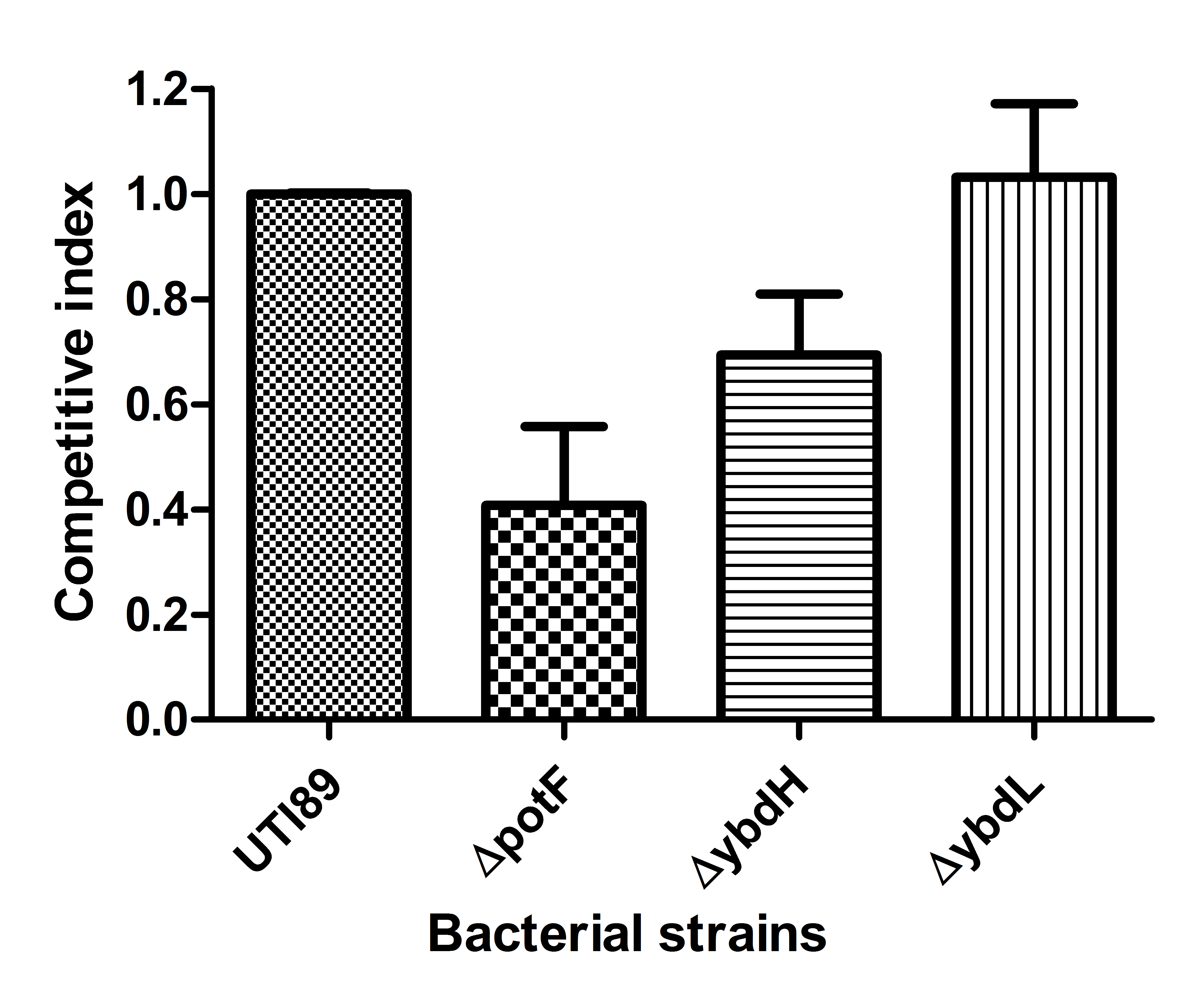

Supplement: Supplementary file 1 — Figure S1. Mutants with no growth defect. The standard error of the mean of two independent 24-h growth experiments is shown. The exact p-value can be seen Additional file 7: Table S3. Unpaired t tests with two-tailed p-values were performed using GraphPad Prism 5 software. (JPG 1007 kb) [file 12866_2017_1008_MOESM1_ESM.jpg]

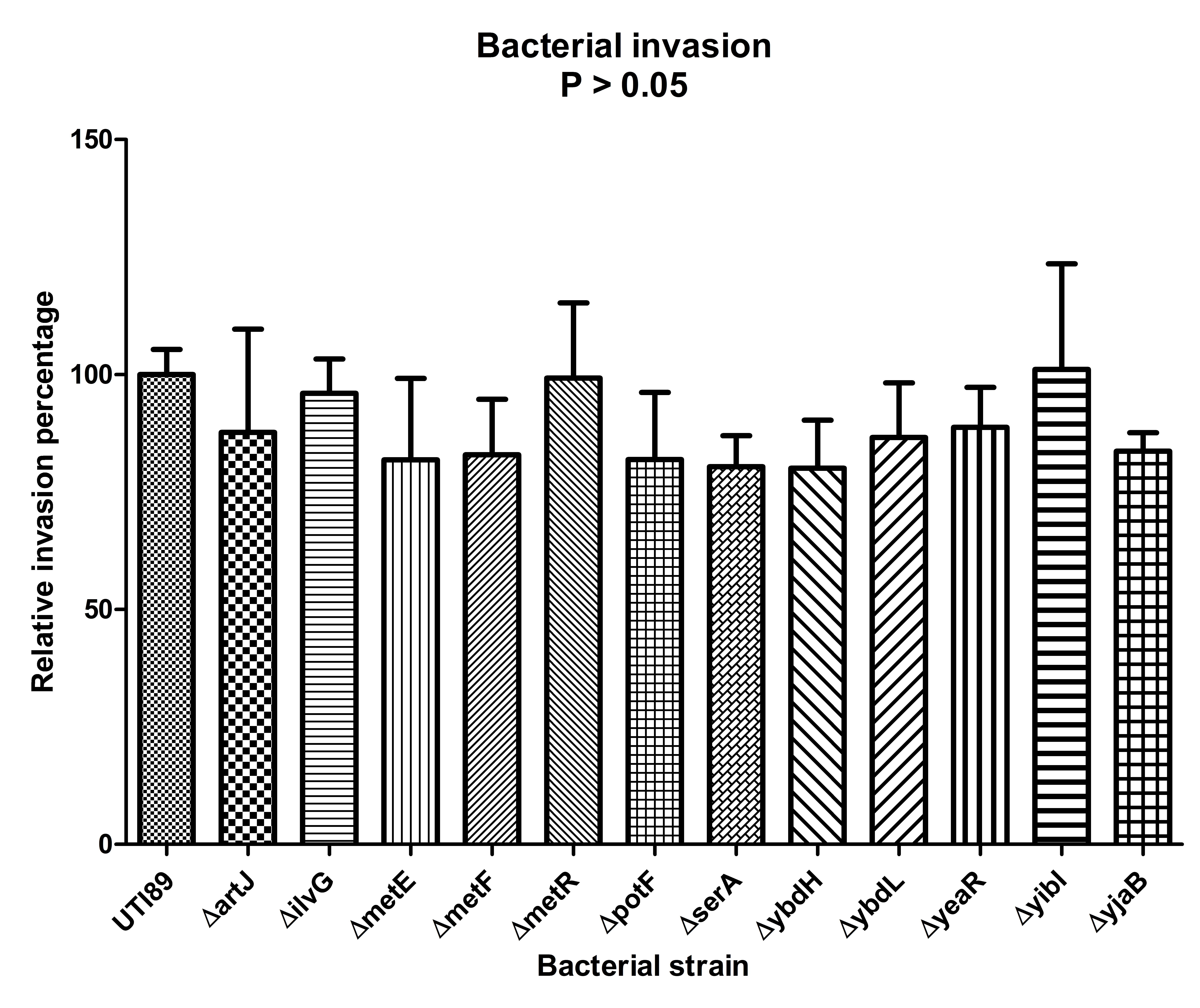

Supplement: Supplementary file 2 — Figure S2. Mutants with no cell invasion defect. The standard error of the mean of three independent invasion assays are shown, P > 0.05 (the exact p-value can be seen in Additional file 8: Table S5). Unpaired t tests with two-tailed p-values were performed using GraphPad Prism 5 software. (JPG 3856 kb) [file 12866_2017_1008_MOESM2_ESM.jpg]

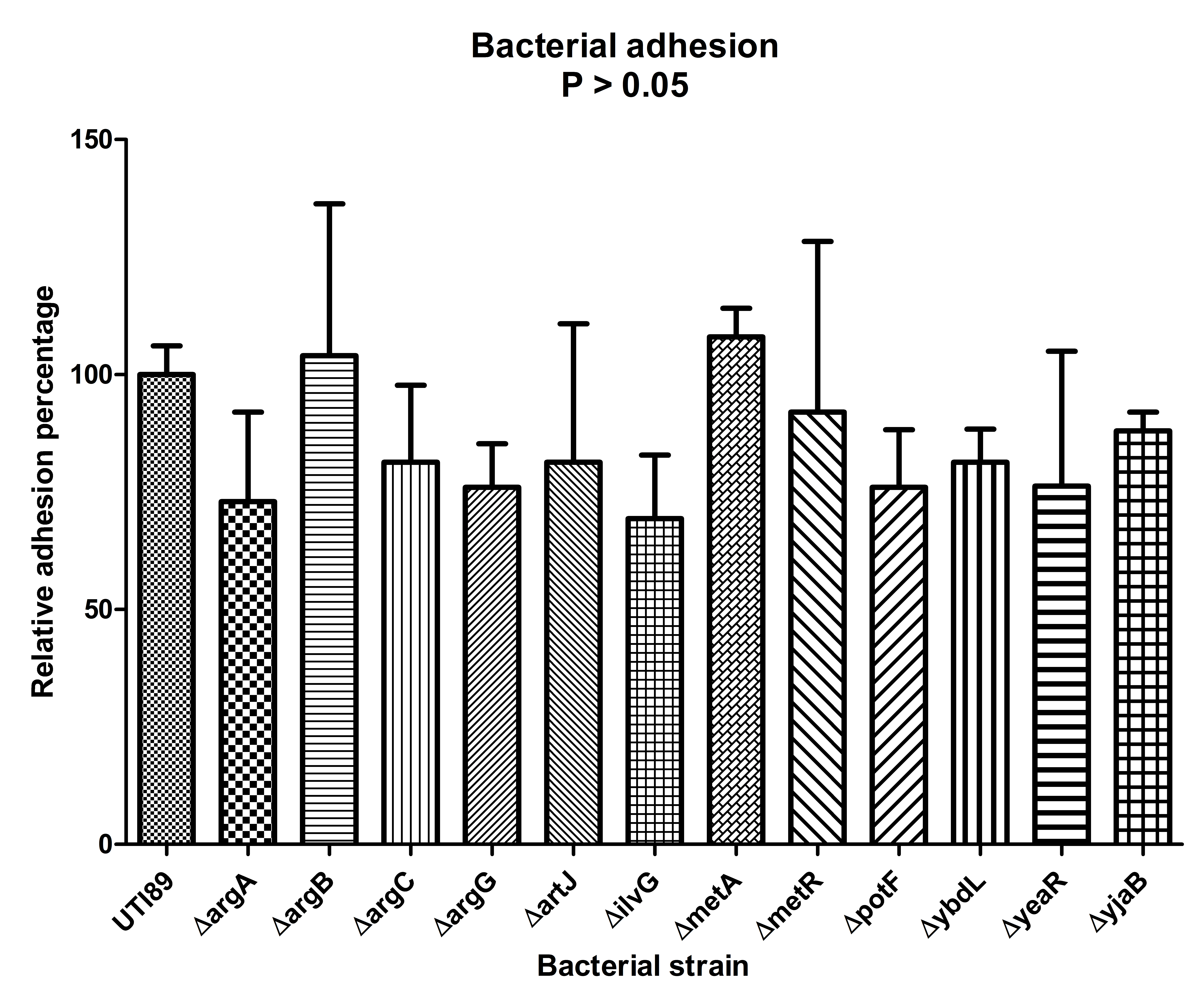

Supplement: Supplementary file 3 — Figure S3. Muatnts with no cell adhesion defect. The standard error of the mean of three independent invasion assays are shown, P > 0.05 (the exact p-value can be seen in Additional file 9: Table S4). Unpaired t tests with two-tailed p-values were performed using GraphPad Prism 5 software. (JPG 3905 kb) [file 12866_2017_1008_MOESM3_ESM.jpg]
