# Supplementary material for: DFI-seq identification of environment-specific gene expression in uropathogenic Escherichia coli
Source: BMC Microbiol. 2017 Apr 24;17:99. doi: 10.1186/s12866-017-1008-4 (PMC5404293; doi:10.1186/s12866-017-1008-4)
Supplement: Supplementary file 4 — Table S6. TMHMM analysis of upregulated hypothetical genes. Gene name of hypothetical genes from Table 5, in parenthesis are the protein BLAST results. (DOCX 13 kb) [file 12866_2017_1008_MOESM4_ESM.docx]

| **Gene name (BLAST results)** | **Uniprot accession number** | **Number of transmembrane helices** |
| --- | --- | --- |
| *UTI89_C4979* (Transposase) | Q1R2N2 | None |
| *UTI89_C4140* | Q1R4Z6 | None |
| *yigZ* | Q1R464 | None |
| *yceI* | Q1RDA2 | None |
| *UTI89_C5140* | Q1R274 | 1 |
| *UTI89_C5136*  (putative membrane protein) | Q1R278 | 4 |
| *UTI89_C5163*  (transcriptional regulator) | Q1R251 | None |
| *UTI89_C5093*  (Phytoene synthase) | Q1R2C1 | None |
| *yfdQ2*  (phage protein) | Q1R2C2 | None |
| *UTI89_C5087*  (phage protein) | Q1R2C7 | None |
| *UTI89_C5088*  (HNH endonuclease) | Q1R2C6 | None |
| *yjjJ*  (transcriptional regulator) | Q1R258 | None |
| *UTI89_C5141* | Q1R273 | None |
| *UTI89_C5086*  (putative membrane protein) | Q1R2C8 | 4 |
| *yecH* | Q1RAN1 | None |
| *yafD*  (EEP domain-containing protein) | Q1RFY2 | None |
| *UTI89_C5108* | Q1R2A6 | None |
| *UTI89_C5078* | Q1R2D6 | None |
| *UTI89_C5146*  (membrane protein) | Q1R268 | 2 |
| *yjjU*  (phospholipase) | Q1R267 | None |
| *UTI89_C3195* | Q1R7M0 | None |
| *yjjM*  (GntR family transcriptional regulator) | Q1R2E8 | None |
