# Supplementary material for: DFI-seq identification of environment-specific gene expression in uropathogenic Escherichia coli
Source: BMC Microbiol. 2017 Apr 24;17:99. doi: 10.1186/s12866-017-1008-4 (PMC5404293; doi:10.1186/s12866-017-1008-4)
Supplement: Supplementary file 5 — Table S7. Primers used for lambda Red recombination. Up and Down primers are used when checking placement of the resistance gene. KO1 and KO1 are used for PCR of the resistance gene before the knockout reaction. (DOCX 15 kb) [file 12866_2017_1008_MOESM5_ESM.docx]

| **Gene** | **Primers** |
| --- | --- |
| argA | **argA up** CTGACTTACGCTCAATAGCCACA  **argA down** GGATAAACGTAATTGCCTGATGC  **argA KO1** AAAACAGAATAAAAATACAATAATTTCGAATAATTATGCAAAGAGGTATACCgtgGTAAAGGAACGTAAAGTGTAGGCTGGAGCTGCTTC  **argA KO2** CCTGATGCGACGCACGAGTGTTACGCATGTCGCATCCGGCGATTTTCATCGCTTACCCTAAATCCGCCATCATATGAATATCCTCCTTA |
| argB | **argB up** TGTATGCCCATAAACCACTGATG  **argB down** GCAGTGAGTCGTTGAATTGTTTG  **argB KO1** CTGAAAGGCGCGGCGGCACAAGCGGTGCAGTGCGCCAATATTCGTTTCGGCTatgCGGAAACGCAGTCTCGTGTAGGCTGGAGCTGCTTC  **argB KO2**  TTCCTTATTTTGAAATTCAATGCCGGAAAGCTGCGCCTCCGGCCAACAAAACTTAAGCTAAAATCCGCGTCATATGAATATCCTCCTTA |
| argC | **argC up** AACCTGGCAACCAGACATAAGAA  **argC down** CTCTTCATCAAGCTGGGTCACTT  **argC KO1**  GAATGTTTTACGTTTAACCTGGCAACCAGACATAAGAAGGTGAATAGCCCCGatgTTGAATACGCTGATTGTGTAGGCTGGAGCTGCTTC  **argC KO2**  ATCCAGCAGTACGCCACCCAGTTTGATAATTAATGGATTCATCATTGCACCCTTAAATAAGAGACTGCGTCATATGAATATCCTCCTTA |
| argE | **argE up** TTCTTATGTCTGGTTGCCAGGTT  **argE down** GCTTTGCTTTTCGTCGTCTTTTA  **argE KO1** TGGTGGGTTTTATTACGCTCAACGTTAGTGTATTTTTATTCATAAATACTGCatgAATATTGATACTATCGTGTAGGCTGGAGCTGCTTC  **argE KO2** TAGGTCGGATAAGATGCGCAAGCATCGCATCCGACATTGTTTGGCCTACGTTTTAATGCCAGCAAAAATGCATATGAATATCCTCCTTA |
| argG | **argG up** GTGGCAAATTCTACTCGTTTTGG  **argG down** CCATGATGTTTTCGATTTTGTGA  **argG KO1** TGAGAGCAGAAATCCAGGCTCATCATCAGTTAATTAAGCAGGGTGTTATTTTatgACGACGATTCTCAAGGTGTAGGCTGGAGCTGCTTC  **argG KO2** ACTGAGGATAAGCAAGGTAACCCCACCCCTGAAGGGCAGGGTTGATGTCGAATTACTGGCCTTTATTTTCCATATGAATATCCTCCTTA |
| artJ | **artJ up** ACCATTACGCTGATGGAAGTGAT  **artJ down** ATGGCACCGCGTTTTATTATCTA  **artJ KO1** TTATTTACTCATATTTATTGCATATAAATTCACTTGATGGCATTGTTATCCCatgCCGCAGACACGGCCAGTGTAGGCTGGAGCTGCTTC  **artJ KO2** CTGACGCTGCTGGTGAAGCAATAAAAAAGCCGCAGGTGCGGCTTTCTGAATCTTACTGTGGGAACCACTGCATATGAATATCCTCCTTA |
| ilvG | **ilvG up** CTACGAGTGATTAGCCTGGTCGT  **ilvG down** AGCCGATACATTGACCTGATGTT  **ilvG KO1** TGAATAACAGCACAAAATTCTGTTTCTCAAGATTCAGGACGGGGAACTAACTatgAATGGCGCACAGTGGGTGTAGGCTGGAGCTGCTTC  **ilvG KO2** CGTTCTAAGGTTTCCGGATTGAAGCGAGCCGATACATTGACCTGATGTTGCATCATGATAATTTCTCCAACATATGAATATCCTCCTTA |
| metA | **metA up** CATATGCTGCCCACTTAACAACA  **metA down** TCGCTTAACGATCGACTATCACA  **metA KO1** CAGCTATCTGGATGTCTAAACGTATAAGCGTATGTAGTGAGGTAATCAGGTTatgCCGATTCGTGTGCCGGTGTAGGCTGGAGCTGCTTC  **metA KO2** AGGTGCCTGAGGTAAGGTGCTGAATCGCTTAACGATCGACTATCACAGAAGATTAATCCAGCGTTGGATTCATATGAATATCCTCCTTA |
| metE | **metE up** GTAACCGCCAATTATGGATGTGT  **metE down** AAAAGGGATACAATCTCGTGCAA  **metE KO1** ATGTGTAAACATCTGGACGGCTAAATACTAAAAATTCCATAAGGGGCATATAatgACAATTCTTAATCACGTGTAGGCTGGAGCTGCTTC  **metE KO2** AGTACTGGAAGCCATTATCGTACTGCAATTTACAATGACTCGTTAATACTCATCATCCCCGACGCAAATTCATATGAATATCCTCCTTA |
| metF | **metF up** GCCAAATCAGATGCCTTAACATC  **metF down** TGTGATTTTCACAAAAGGCACAC  **metF KO1** GATAGATGTGCACAACACAACATATAACGATAAGCGATTGATGAGGTAAGGTatgAGCTTTTTTCACGCCGTGTAGGCTGGAGCTGCTTC  **metF KO2** GCTCTGTTTAAAATTTGTGATCACTGTGTGATTTTCACAAAAGGCACACTATTTATAAACCAGGTCGAACCATATGAATATCCTCCTTA |
| metR | **metR up** ACACATCCATAATTGGCGGTTAC  **metR down** CATTTCTGGACTGGCTTATTGCT  **metR KO1** TTACTGTATATTCCTCAAGCGCAAATTATTCATGCCGAAGTGAAGGACTTTCatgATCGAAGTAAAACACGTGTAGGCTGGAGCTGCTTC  **metR KO2**  CTGCTGGTAAACCTGGCTATCTGGCACCAACAGCCGCACTGGCCAACGTTTATTACAGGCGCGCTGGTGACATATGAATATCCTCCTTA |
| potF | **potF up** CTGACGATGAAGGGCAAAATAAC  **potF down** AGGATTTGGTCAGGTTACGGATT  **potF KO1** CGTTTTTTAATCCGAGCTATAGTCTCAAACCCTGGCTAAAGTTATTCTTGCGatgCTTTTATATAGTGAGGTGTAGGCTGGAGCTGCTTC  **potF KO2** GTGCGAATTGCCGGCGGGTGTGGTGCGCCCGTCCGGCATCTACGGCTGCGGATTATTTTCCGCTCTTCACCATATGAATATCCTCCTTA |
| serA | **serA up** GTGTCACGTTTTTACCTGGCAAT  **serA down** ACAACGCATTGATCTGACTTTGA  **serA KO1** GAAAGGCGGATGCAAATCCGCACACAACATTTCAAAAGACAGGATTGGGTAAatgGCAAAGGTATCGCTGGTGTAGGCTGGAGCTGCTTC  **serA KO2** TAAAAAAACGGGCAAGTCAGTGACCTGCCCGTTAATTTTCAGAGAAGGGGAATTAGTACAGCAGACGGGCCATATGAATATCCTCCTTA |
| ybdH | **ybdH up** GTGGAAGTTTGCTTTGTGGAATC  **ybdH down** CCTCTTAATTCAGGAGGGCATTT  **ybdH KO1** TTGGCAATCAAGACGTTTAGATGTCTAAATAAAACAATAAGGACAACACAACatgCCTCACAATCCTATCGTGTAGGCTGGAGCTGCTTC  **ybdH KO2** GGACGCGCGCTACGGTGGAAAAGGCGGCGCGCGCTGCTGCTAAATTTGTACGTCAGGCTTTAAACGATTCCATATGAATATCCTCCTTA |
| ybdL | **ybdL up** AATCGTGCAGGTGATTGAAACTT  **ybdL down** CAGTTGGGATGGGACGATAATAA  **ybdL KO1** TATAGTGCCTTCAACACGCAACCAACACGCAACTTCGTCAGGTACAATAAAAatgACAAATAACCCTCTGGTGTAGGCTGGAGCTGCTTC  **ybdL KO2** GCAAGAGTTGTTTGCCGACCGTCAATTTTCCCGCGCCTGGACGGTTAAATAACTACAGCTGGCGCAGGCGCATATGAATATCCTCCTTA |
| yeaR | **yeaR up** GATTCTTCTCTGATCCGGTGCTA  **yeaR down** CATGTTCGTTTTCCGTGTCATAA  **yeaR KO1** GAAACGGCTGGAATAAACCGTTTTCAGCGCATTCACCGAAGGAGGGAAAAGGatgCTGCAAATCCCACAGGTGTAGGCTGGAGCTGCTTC  **yeaR KO2** CGCCATTGCTGTTATTGGTGACGGTCACGGTATACGTTGCTTTGCCCATGATTCATTTCCCGTTATGAATCATATGAATATCCTCCTTA |
| yibI | **yibI up** TTCTTTGGTGTTATGGCGAGAAT  **yibI down** GCGCCACGTAAGTTAAAACAATC  **yibI KO1** CGTGAATCCGAGGCAGATatgTTTCTAAACTATTTCGCGTTGGGAGTGCTGATCTTCGTCTTTCTGGTGAGTGTAGGCTGGAGCTGCTTC  **yibI KO2** CAATCAATAGATCCATAATTAGATCTCCGGAAACGTGGAATAGTCAGTCTTTTTCTCGGCGGCGAGTTGGCATATGAATATCCTCCTTA |
| yjaB | **yjaB up** TCTTCGGCATCAGGTTAAGGATA  **yjaB down** CATTGGTGGGTACGAAACCTAAA  **yjaB KO1**  GTTTCAAGGAAAAGGCTACGTTAGAATATAAGAATGACGAAAAGGAGAGAAAatgGTTATTAGTATTCGAGTGTAGGCTGGAGCTGCTTC  **yjaB KO2** TTGGTGGGTACGAAACCTAAAGTTCAGCCCACCGGGATGAGAAAAAAACCGCCTACACCCCCACATATGCCATATGAATATCCTCCTTA |
| UTI89_C5139 | **UTI89_C5139 up** AGTACAACAGGCTCACGAAGACC  **UTI89_C5139 down** AAGTTGTAGGTTCCGCAATGAAA  **UTI89_C5139 KO1**  TCCCCGTCATGGGGTGACAGAGGGATAAAGTTCAATTTCTCTTGCGCGTGAAatgCGTAAAATCAGTAACGTGTAGGCTGGAGCTGCTTC  **UTI89_C5139 KO2**  TTTGTGCTGCGGCTGCGCAGCTATTGATTGGTCTTTTTACCGGGAACTGGGCTTACGGGGCGATAGCCGGCATATGAATATCCTCCTTA |
