# Supplementary material for: DFI-seq identification of environment-specific gene expression in uropathogenic Escherichia coli
Source: BMC Microbiol. 2017 Apr 24;17:99. doi: 10.1186/s12866-017-1008-4 (PMC5404293; doi:10.1186/s12866-017-1008-4)
Supplement: Supplementary file 6 — Table S8. Primers used for quantitative real time RT-PCR (DOCX 15 kb) [file 12866_2017_1008_MOESM6_ESM.docx]

| **Sequence** | **Description** |
| --- | --- |
| GTCGAACGGTAACAGGAAGAAG | rrsA fw |
| GGCAGTTTCCCAGACATTACTC | rrsA rv |
| Acccaactaacgaccgtctg | rpoB fwd |
| Accggaaccttcgatttctt | rpoB rev |
| GTTCCCTCACCACATTACGATT | ampD UTI89 fwd |
| CTAATATTGTGCACCACCAGCA | ampD UTI89 rev |
| AAGCCATTGAGCATGAGAATTT | argA UTI89 fwd |
| GTGCAACAACCCGATATCATTA | argA UTI89 rev |
| TTAACGATGTCGCCTTCTATGA | argC UTI89 fwd |
| GGTATTGATGGGTAAAGCCGTA | argC UTI89 rev |
| TTATGCTCCGGCAGAGTTTATT | argD UTI89 fwd |
| ACATACTCCTTGCCCTGTTGAT | argD UTI89 rev |
| AACAAATTACCGCCATTTATCG | argE UTI89 fwd |
| TTATTGAAGGTGTGGCAATCAG | argE UTI89 rev |
| TGGACTGACCTATTTCAACACG | argG UTI89 fwd |
| TCTTCTTTCATAGCAGCAACCA | argG UTI89 rev |
| TTACTAATCACGCGTTCGACAG | artJ UTI89 fwd |
| CGGCGTCATATTTTCTGAATTT | artJ UTI89 rev |
| CAGTTCGAAGTGGTTCATTCAC | asnA UTI89 fwd |
| CCTAAGGTCTGACGTTTCCACT | asnA UTI89 rev |
| AAAACTCATGTGCTGGCAGTAA | asnB UTI89 fwd |
| ACTGGTAACGATCGCCATATTC | asnB UTI89 rev |
| CTAGGCCCGTATATTCGTGAAG | crl UTI89 fwd |
| GCCAGACAATCGAAAAAGAATC | crl UTI89 rev |
| TTATCGTTCTTGTTTTGCTTGC | ecnA UTI89 fwd |
| TTGCCGAGGTGTTTGATATCTT | ecnA UTI89 rev |
| TATTAACTGCCTGCAACACCAC | ecnB UTI89 fwd |
| CCATCAGAAATGTCTTCACCAA | ecnB UTI89 rev |
| TGCTGATATCACCGAATTTTTG | fadB UTI89 fwd |
| AACTGTTCTTCAGGAACGAGGA | fadB UTI89 rev |
| AAGGGGTTATCAGCCAGTTACA | fliE UTI89 fwd |
| GCGACTGTTTGTGTATCGCTTA | fliE UTI89 rev |
| AATCCGAAAATTCCATTGATTG | fliF UTI89 fwd |
| GATTGCTGAATAATGTGCGGTA | fliF UTI89 rev |
| CTGTGAGTGAGCTGGATTATCG | galS UTI89 fwd |
| CCAATGGTATCGCTAACCTGAG | galS UTI89 rev |
| GCAAATAAATTGCACTCAGCAG | hlyA UTI89 fwd |
| CAAGGTCATTAAGGCTTGAACC | hlyA UTI89 rev |
| AATGTTGACGTTGCAGAAATTG | ilvC UTI89 fwd |
| GGAAATTTATTGCGGAAATTGA | ilvC UTI89 rev |
| GGATATTCTGGGGTTGTCGTTA | ilvG UTI89 fwd |
| GATACGCGGTAACTCTTCCAGT | ilvG UTI89 rev |
| TTAAACTCACGACTGGTGATGC | ilvY UTI89 fwd |
| GCCAGTGACTACCTTTTCCATC | ilvY UTI89 rev |
| CAGAGTATGCCGGTGTCTCTTA | lacI UTI89 fwd |
| GGTTGGGAATGTAATTCAGCTC | lacI UTI89 rev |
| AAAGTGATATTCGTGGCGTCTT | manZ UTI89 fwd |
| GAAGTTCCATGAACCCTGAAAG | manZ UTI89 rev |
| TACGTCTCAATGACGAGCAACT | menE UTI89 fwd |
| CAAAGCTGGAGGCTAATTCATC | menE UTI89 rev |
| TTAACCTGATGCCGAAGAAGAT | metA UTI89 fwd |
| ATATCGACCTGCAAAGGTGAGT | metA UTI89 rev |
| TCTGCTTTATGCCAATTCTTCA | metC UTI89 fwd |
| TCGGGATTCCTGTTTTTATACG | metC UTI89 rev |
| TTCTTAATCACACCCTCGGTTT | metE UTI89 fwd |
| CCAATAACTCTCTTGCGCTTTT | metE UTI89 rev |
| GCCTGAAACCGAAGTTTGTATC | metF UTI89 fwd |
| GCCTTTAATAATGCTGTGCGTA | metF UTI89 rev |
| ACGCCTGTACGGTAAACTATGC | metJ UTI89 fwd |
| AGACCAGACCACACAGTGAATG | metJ UTI89 rev |
| AACACCTGAAAACGCTACAAGC | metR UTI89 fwd |
| CGTTGTTCCAGATCGCTAAACT | metR UTI89 rev |
| GCTGGGTTGAAGAGGTGTTTAT | nadC UTI89 fwd |
| GTCATCCACATGCCAGATTATG | nadC UTI89 rev |
| CGGTTTTATCGATGTGCAGTTA | nagA UTI89 fwd |
| ATTTCTCATTGGCTTTCTGCAT | nagA UTI89 rev |
| CTTCAAACAGAAGGGTGAAACC | narG UTI89 fwd |
| CAGTCACGGTTGGTATTGAGAA | narG UTI89 rev |
| TTCAAGCTGGATGCGTTACTTA | pepQ UTI89 fwd |
| ATAGGGATGATCGTCGAGAAAA | pepQ UTI89 rev |
| TGGCCAACTTTGAAAAAGAAAC | potF UTI89 fwd |
| GCCATTAATTTGCCTTCCAGTA | potF UTI89 rev |
| AGCAGTTTGATCGCAAAGGTAT | purC UTI89 fwd |
| ATCCAGCTTTTTCACCAGACAT | purC UTI89 rev |
| CGCTGGATGATGAACAATTAAA | serA UTI89 fwd |
| CACGTCTTCAGTCAGATGGGTA | serA UTI89 rev |
| AATAACACGCCATTTCCTTTGT | UTI89_C0254 fwd |
| TGAAACTCAGCAAGATCAGCAT | UTI89_C0254 rev |
| TAAACAGCTTAGCGAACAGCAA | UTI89_C0374 fwd |
| TGCTTCACCTGGGCTTTATATT | UTI89_C0374 rev |
| CGGAGAGACAAACGGTTTAAGT | UTI89_C1129 fwd |
| AGTTTTACCAAACCAGGTGAGC | UTI89_C1129 rev |
| TGTTCTCCGCTATAACCTCCAT | UTI89_C2260 fwd |
| CTTGACAGACAGGCTACATTGC | UTI89_C2260 rev |
| GCATTTATACTTTGCCGATTCC | UTI89_C3136 fwd |
| TTTGTGCGCATTCATAAAATTC | UTI89_C3136 rev |
| TTGGAGACAGGAGATGTAACGA | UTI89_C4885 fwd |
| CGGAGGTAAAATCATGGACTTC | UTI89_C4885 rev |
| tgGAACTCATTGAAAAACATGC | yaiM UTI89 fwd |
| ATTGGGAATAATGCCGATACAC | yaiM UTI89 rev |
| TTTAGCTCACCTGCATTTAGCC | yajB UTI89 fwd |
| ACGAAATCAGCCAGTAAATTGC | yajB UTI89 rev |
| GTCTATAGTGCGGGTGACAGTG | ybaS UTI89 fwd |
| GCAAGGGCTAACGTACAGACTT | ybaS UTI89 rev |
| CCAGGGGTAAAGCATATTTTGT | ybdH UTI89 fwd |
| CCAGTTGTTGTACATCGCTTTC | ybdH UTI89 rev |
| TAACCCTCTGATTCCACAAAGC | ybdL UTI89 fwd |
| GGTGAAAATAGTGGTGCCAAGT | ybdL UTI89 rev |
| ACGCCCGTACATGTCTCTCTAT | ydcX UTI89 fwd |
| CAGGTGATAAGAAATGCGATTG | ydcX UTI89 rev |
| CGCCTTTCTGGAATAAACAAAC | yeaR UTI89 fwd |
| TCCTTTATCAAGATGACGTTCG | yeaR UTI89 rev |
| TAAACTATTTCGCGTTGGGAGT | yibI UTI89 fwd |
| ATGTCATGTATCGCGATGATTC | yibI UTI89 rev |
| TCTGGTGAGTGGTTTGATTTTG | yibH UTI89 fwd |
| AGTAACAATTCCCGTCACCTGT | yibH UTI89 rev |
| GATATGAATATCCGGCGAAAAA | yifB UTI89 fwd |
| AATCATATCGTCCCCCTTCTTT | yifB UTI89 rev |
| AAATCCAGGTTGATGGTGAGTT | yiiM UTI89 fwd |
| GCTCACGAGGATAATGACACAG | yiiM UTI89 rev |
| ATGTTGTTAAGTGGGCAGCATA | yjaB UTI89 fwd |
| GCACATCAGGATCGATAAACAG | yjaB UTI89 rev |
| GCAAAGAAGAAGGCGGTATCTA | yjeM UTI89 fwd |
| CGTGGTCCGACACTATTATTCA | yjeM UTI89 rev |
| AGTGATGCTGAAGCGTTTCTTT | ynfK UTI89 fwd |
| CCGTTTTCCCTACAGAAGTGTC | ynfK UTI89 rev |
