# Supplementary material for: DFI-seq identification of environment-specific gene expression in uropathogenic Escherichia coli
Source: BMC Microbiol. 2017 Apr 24;17:99. doi: 10.1186/s12866-017-1008-4 (PMC5404293; doi:10.1186/s12866-017-1008-4)
Supplement: Supplementary file 7 — Table S3. Competitive index p-values. (DOCX 12 kb) [file 12866_2017_1008_MOESM7_ESM.docx]

| **Strain** | **P-value** |
| --- | --- |
| UTI89Δ*argA* | <0.0001 |
| UTI89Δ*argB* | <0.0001 |
| UTI89Δ*argC* | 0.0020 |
| UTI89Δ*argE* | 0.0008 |
| UTI89Δ*argG* | <0.0001 |
| UTI89Δ*artJ* | 0.0018 |
| UTI89Δ*ilvG* | 0.0422 |
| UTI89Δ*metA* | 0.0002 |
| UTI89Δ*metE* | <0.0001 |
| UTI89Δ*metF* | <0.0001 |
| UTI89Δ*metR* | 0.0005 |
| UTI89Δ*potF* | 0.0583 |
| UTI89Δ*serA* | 0.0015 |
| UTI89Δ*ybdH* | 0.1185 |
| UTI89Δ*ybdL* | 0.8405 |
| UTI89Δ*yeaR* | 0.0009 |
| UTI89Δ*yibI* | 0.0083 |
| UTI89Δ*yjaB* | 0.0131 |
