# Supplementary material for: DFI-seq identification of environment-specific gene expression in uropathogenic Escherichia coli
Source: BMC Microbiol. 2017 Apr 24;17:99. doi: 10.1186/s12866-017-1008-4 (PMC5404293; doi:10.1186/s12866-017-1008-4)
Supplement: Supplementary file 8 — Table S5. P-values for cell invasion assays. (DOCX 12 kb) [file 12866_2017_1008_MOESM8_ESM.docx]

| **Strain** | **P-value** |
| --- | --- |
| DH5α | <0.0001 |
| UTI89Δ*argA* | 0.0428 |
| UTI89Δ*argB* | 0.0012 |
| UTI89Δ*argC* | 0.0036 |
| UTI89Δ*argE* | 0.0285 |
| UTI89Δ*argG* | 0.0053 |
| UTI89Δ*artJ* | 0.6149 |
| UTI89Δ*ilvG* | 0.6801 |
| UTI89Δ*metA* | 0.0482 |
| UTI89Δ*metE* | 0.3738 |
| UTI89Δ*metF* | 0.2571 |
| UTI89Δ*metR* | 0.9663 |
| UTI89Δ*potF* | 0.3005 |
| UTI89Δ*serA* | 0.0818 |
| UTI89Δ*ybdH* | 0.1591 |
| UTI89Δ*ybdL* | 0.3537 |
| UTI89Δ*yeaR* | 0.3262 |
| UTI89Δ*yibI* | 0.9648 |
| UTI89Δ*yjaB* | 0.0702 |
