# Supplementary material for: DFI-seq identification of environment-specific gene expression in uropathogenic Escherichia coli
Source: BMC Microbiol. 2017 Apr 24;17:99. doi: 10.1186/s12866-017-1008-4 (PMC5404293; doi:10.1186/s12866-017-1008-4)
Supplement: Supplementary file 9 — Table S4. P-values for cell adhesion assays. (DOCX 12 kb) [file 12866_2017_1008_MOESM9_ESM.docx]

| **Strain** | **P-value** |
| --- | --- |
| DH5α | 0.0002 |
| UTI89Δ*argA* | 0.2478 |
| UTI89Δ*argB* | 0.9091 |
| UTI89Δ*argC* | 0.3459 |
| UTI89Δ*argE* | 0.0051 |
| UTI89Δ*argG* | 0.0961 |
| UTI89Δ*artJ* | 0.5681 |
| UTI89Δ*ilvG* | 0.1078 |
| UTI89Δ*metA* | 0.4069 |
| UTI89Δ*metE* | 0.0273 |
| UTI89Δ*metF* | 0.0354 |
| UTI89Δ*metR* | 0.8386 |
| UTI89Δ*potF* | 0.1538 |
| UTI89Δ*serA* | 0.0306 |
| UTI89Δ*ybdH* | 0.0032 |
| UTI89Δ*ybdL* | 0.1161 |
| UTI89Δ*yeaR* | 0.4639 |
| UTI89Δ*yibI* | 0.0383 |
| UTI89Δ*yjaB* | 0.1757 |
