# Supplementary material for: DFI-seq identification of environment-specific gene expression in uropathogenic Escherichia coli
Source: BMC Microbiol. 2017 Apr 24;17:99. doi: 10.1186/s12866-017-1008-4 (PMC5404293; doi:10.1186/s12866-017-1008-4)
Supplement: Supplementary file 10 — Table S1. P-values for RT-qPCR verification of genes identified by DFI. (DOCX 12 kb) [file 12866_2017_1008_MOESM10_ESM.docx]

| **Gene** | **P-value** |
| --- | --- |
| *argA* | 0,072 |
| *argC* | 0,002 |
| *argE* | 0,004 |
| *argG* | 0,002 |
| *artJ* | 0,002 |
| *asnA* | 0,492 |
| *asnB* | 0,001 |
| *ecnA* | 0,001 |
| *ecnB* | 0,046 |
| *ilvC* | 0,699 |
| *ilvG* | 0,002 |
| *manZ* | 0,001 |
| *metA* | 0,002 |
| *metC* | 0,001 |
| *metE* | 0,002 |
| *metF* | 0,002 |
| *potF* | 0,002 |
| *serA* | 0,046 |
| *UTI89_C1129* | 0,492 |
| *UTI89_C4885* | 0,027 |
| *yaiM* | 0,001 |
| *yajB* | 0,04 |
| *ybdH* | 0,002 |
| *ybdL* | 0,002 |
| *yeaR* | 0,046 |
| *yibI* | 0,002 |
