# Supplementary material for: DFI-seq identification of environment-specific gene expression in uropathogenic Escherichia coli
Source: BMC Microbiol. 2017 Apr 24;17:99. doi: 10.1186/s12866-017-1008-4 (PMC5404293; doi:10.1186/s12866-017-1008-4)
Supplement: Supplementary file 11 — Table S2. P-values for RT-qPCR verification of genes identified by DFI-seq. (DOCX 13 kb) [file 12866_2017_1008_MOESM11_ESM.docx]

| **Gene** | **P-value** |
| --- | --- |
| *ampD* | 0,892 |
| *argC* | 0,002 |
| *argD* | 0,794 |
| *argE* | 0,004 |
| *argG* | 0,002 |
| *artJ* | 0,002 |
| *Crl* | 0,77 |
| *fadB* | 0,029 |
| *fliE* | 0,001 |
| *fliF* | 0,001 |
| *galS* | 0,26 |
| *hlyA* | 0,015 |
| *ilvG* | 0,002 |
| *ilvY* | 0,087 |
| *lacI* | 0,001 |
| *menE* | 0,997 |
| *metA* | 0,002 |
| *metE* | 0,002 |
| *metF* | 0,002 |
| *metJ* | 0,409 |
| *metR* | 0,002 |
| *nadC* | 0,404 |
| *nagA* | 0,011 |
| *narG* | 0,034 |
| *pepQ* | 0,355 |
| *potF* | 0,002 |
| *purC* | 0,063 |
| *UTI89_C0254* | 0,136 |
| *UTI89_C0374* | 0,001 |
| *UTI89_C2260* | 0,006 |
| *UTI89_C3136* | 0,576 |
| *ybaS* | 0,002 |
| *ybdH* | 0,002 |
| *ybdL* | 0,002 |
| *ydcX* | 0,002 |
| *yibH* | 0,002 |
| *yibI* | 0,002 |
| *yifB* | 0,053 |
| *yiiM* | 0,001 |
| *yjaB* | 0,477 |
| *yjeM* | 0,001 |
| *ynfK* | 0,496 |
